# Combined Immune‐Peptide Nanofiber with HSP70/AKT/mTOR Axis Blockade Enhances Photothermal Immunotherapy, Inhibiting Tumor Growth and Recurrence

**DOI:** 10.1002/advs.78434

**Published:** 2026-10-25

**Authors:** Xinyu Zhang, Zhenqi Jiang, Wenjia Zhang, Juncheng Wu, Haozhuo Guo, Changlei Li, Mingwei Sun, Xiaoying Tang, Yue Zhang, Wei Huang, Huadan Xue, Yang Du

**Affiliations:** ^1^ Department of Radiology State Key Laboratory of Complex Severe and Rare Diseases Peking Union Medical College Hospital Peking Union Medical College and Chinese Academy of Medical Sciences Beijing China; ^2^ CAS Key Laboratory of Molecular Imaging Beijing Key Laboratory of Molecular Imaging Institute of Automation Chinese Academy of Sciences Beijing China; ^3^ School Medical Science and Engineering Beijing Institute of Technology Zhengzhou Academy of Intelligent Technology Beijing Institute of Technology Beijing China; ^4^ Department of Radiology Peking University People's Hospital Beijing China; ^5^ Department of Interventional Radiology Shengjing Hospital of China Medical University Shenyang Liaoning China; ^6^ Laboratory Animal Research Facility National Infrastructures for Translational Medicine Institute of Clinical Medicine Peking Union Medical College Hospital Chinese Academy of Medical Sciences & Peking Union Medical College Beijing China; ^7^ Department of Radiation Oncology Shandong Cancer Hospital and Institute Shandong First Medical University Shandong Academy of Medical Sciences Jinan Shandong China; ^8^ University of Chinese Academy of Sciences Beijing China

**Keywords:** “cold” tumors, carrier‐free self‐assembled nanofibers, HSP70/AKT/mTOR signaling pathway, photothermal immunotherapy (PTIT), tumor resistance and recurrence

## Abstract

Although immune checkpoint blockade (ICB) therapies have shown clinical benefit, ICBs exert limited effects on most “immune‐cold” solid tumors, i.e., tumors with low immune infiltration. Photothermal immunotherapy (PTIT) converts “cold” tumors into “hot” tumors, thereby enhancing immune responses and improving ICB efficacy. Hence, developing a strategy that can integrate PTIT and ICB treatment is desirable. In this study, we designed a unique carrier‐free PSP@IR‐CF_3_ nanofiber, self‐assembled from the programmed death‐ligand 1 (PD‐L1) targeting immune‐peptide NTGYFYGDQ (PSP) and a PTIT agent (IR‐CF_3_). The nanofibers enable precise tumor targeting and immune checkpoint inhibition by specifically binding to PD‐L1 on the tumor cell surface, also providing an NIR‐mediated photothermal effect. Combining PSP@IR‐CF_3_ nanofibers with NIR induced mild PTIT, which effectively activated the tumor immune microenvironment and suppressed tumor growth with lower immunotoxicity. Additionally, we identified activation of the heat shock protein 70 (HSP70) /AKT/mTOR axis following PTIT, suggesting a potential role in post‐treatment tumor survival and recurrence. By incorporating the mTOR inhibitor rapamycin, the combination treatment further enhanced therapeutic efficacy and reduced tumor recurrence.

## Graphical abstract

A carrier‐free peptide‐guided PSP@IR‐CF_3_ nanofiber system integrates PD‐L1 targeting and photothermal immunotherapy for enhanced cancer treatment. By revealing PTIT‐induced activation of the HSP70/AKT/mTOR pathway and incorporating mTOR inhibition, this strategy effectively overcomes therapeutic resistance and prevents tumor recurrence, providing a promising avenue for durable antitumor immunity.

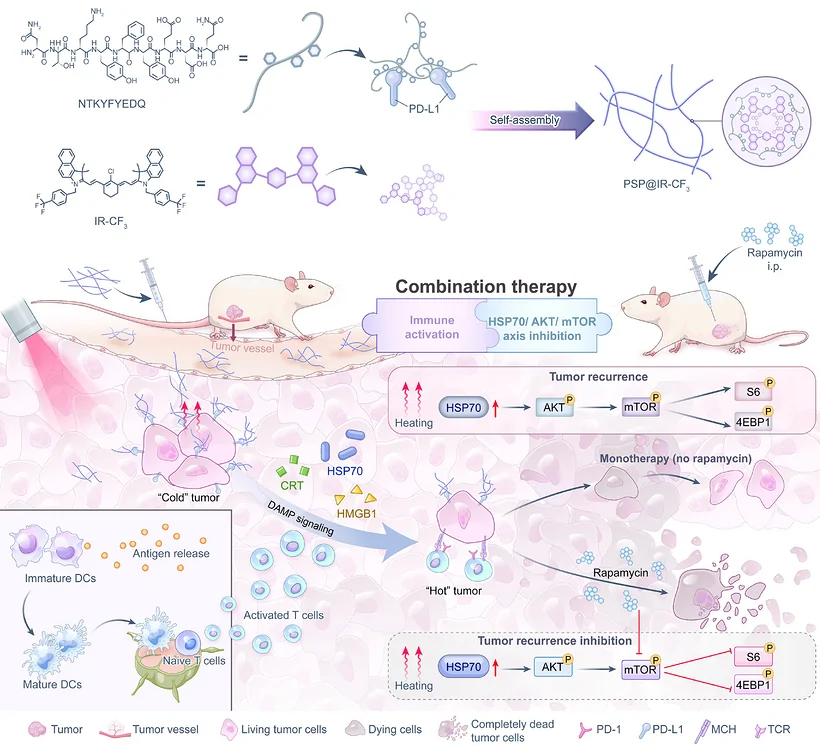

## 1 Introduction

“Cold” tumors, characterized by low immune infiltration and poor responsiveness to immunotherapy, represent a major challenge in immunotherapy [1, 2]. For example, colorectal cancer exhibits an immunologically “cold” subtype, typically lacking immune cell infiltration and presenting an immunosuppressive microenvironment, making it difficult to induce an immune response and reducing the efficacy of immune checkpoint blockade (ICB) therapy [3, 4, 5, 6, 7]. Photothermal immunotherapy (PTIT) is increasingly being recognized for its precision and non‐invasiveness, particularly in the treatment of immunologically “cold” tumors [1, 8, 9, 10, 11]. PTIT can induce immunogenic cell death (ICD), a process that releases key tumor‐associated antigens and damage‐associated molecular patterns (DAMPs), converting the tumor site into an “in situ vaccine” [12, 13]. Immune cells, such as dendritic cells (DCs) and macrophages, capture and process these antigens and DAMPs, promoting effective, targeted adaptive immune responses against various solid tumors [14]. However, despite its promising efficacy, PTIT has certain limitations. It often fails to completely inhibit tumor growth and metastasis [15, 16]. This is partially due to inherent resistance mechanisms in the tumor microenvironment (TME), particularly through the upregulation of programmed cell death ligand‐1 (PD‐L1) under local immune activation and interferon stimulation, which may contribute to immune escape and reduce the effectiveness of PTIT in cancer treatment [17, 18]. Therefore, combining PTIT with ICB therapies, such as inhibitors targeting PD‐L1 or CTLA‐4, has shown great potential in both preclinical and early clinical studies [17, 19].

Currently, the combination of PTIT and immune checkpoint inhibitors (ICIs) typically follows a sequential protocol [20]. However, this approach may lead to complications due to intervals between treatments. After PTIT, PD‐L1 expression may rapidly increase in the TME, triggering immune escape and leading to tumor recurrence or metastasis, thereby impairing the efficacy of subsequent ICIs [20]. Additionally, frequent dosing may reduce patient compliance. To overcome these challenges, the co‐delivery of PTIT agents and ICIs using carrier‐mediated drug delivery systems is being explored [21, 22, 23]. Although this strategy has synergistic potential, challenges such as nanocarrier toxicity and uncontrolled drug release may lead to variable treatment outcomes [24].

Recent studies have shown that the self‐assembly of bioactive components can facilitate carrier‐free delivery [24, 25]. This approach enables synergistic effects with a single dose, simplifying clinical procedures, and improving treatment feasibility and patient compliance [26, 27]. Li S. et al. constructed supramolecular nanofibrils by coassembling the clinically approved immunomodulatory peptide TP5 with the photothermal dye indocyanine green, achieving a carrier‐free photothermal‐immune synergistic system capable of enhancing antitumor immunity following localized photothermal therapy [27]. This strategy demonstrated remarkable therapeutic effects and prolonged survival in tumor‐bearing mouse models. These developments highlight the potential of carrier‐free photosensitizer‐based nanoparticles in advanced immunotherapeutic strategies. However, such studies often rely on passive delivery, lacking the precision of active targeting strategies that leverage ligand‐receptor interactions for drug‐specific targeting of cancer cells, which increases off‐target effects, reduces therapeutic efficacy, and elevates the risk of side effects [28, 29, 30]. Hence, peptides with targeting abilities, immunotherapeutic effects, and self‐assembly properties are attracting increasing attention [31, 32]. NTGYFYGDQ (PSP), a PD‐L1 targeting immune peptide, possesses dual functionality: it can self‐assemble into nanofibers and simultaneously block immune checkpoints. PSP markedly enhances immune cell recognition and antitumor immune responses by disrupting tumor immune evasion mechanisms [33].

Hence, in this study, we aimed to develop a novel carrier‐free PSP@IR‐CF_3_ nanofiber system through self‐assembling PSP with the mild PTIT agent IR‐CF_3_. The specific interaction between PSP and PD‐L1 significantly enhances precise tumor targeting and ICB efficacy, and IR‐CF_3_ converts light energy into heat under NIR irradiation. Consequently, the unique design of PSP@IR‐CF_3_ nanofibers exhibits multifunctional theranostic properties and possesses considerable potential for clinical applications. Although PTIT activates antitumor immunity, adaptive responses of residual tumor cells to treatment‐induced stress may contribute to tumor recurrence. Stress‐responsive pathways, such as the PI3K/AKT/mTOR pathway, are known to regulate tumor cell survival under therapeutic pressure [34]. Therefore, we performed a transcriptomic analysis and identified activation of the heat shock protein 70 (HSP70) /AKT/mTOR signaling axis as a potential mediator associated with therapeutic resistance and tumor recurrence. To investigate the therapeutic relevance of this finding, we used rapamycin, a specific mTOR inhibitor approved by the U.S. Food and Drug Administration (FDA) for certain cancer therapies [35, 36, 37, 38, 39]. The combination of PSP@IR‐CF_3_‐mediated PTIT with rapamycin significantly reduced tumor recurrence and improved long‐term therapeutic efficacy, supporting the potential of mTOR pathway inhibition as a strategy to attenuate PTIT‐associated adaptive resistance.

Overall, this study presents a carrier‐free peptide‐guided nanoplatform that synergizes ICB and PTIT, while subsequent mTOR inhibition may help alleviate adaptive resistance and tumor recurrence, offering a promising strategy for durable cancer therapy (Figure 1).

**FIGURE 1:**
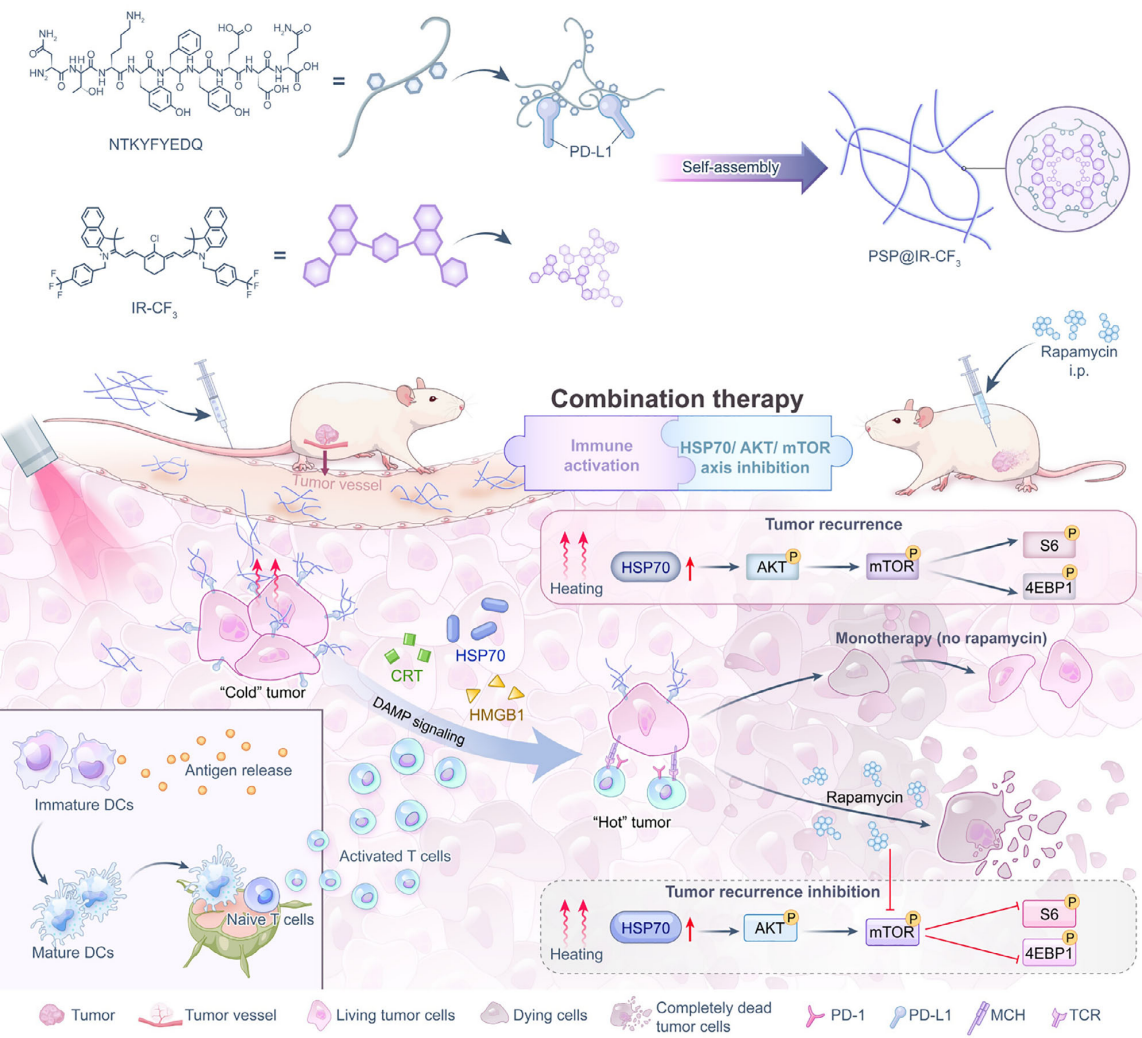
Schematic illustration of the development of PSP@IR‐CF_3_ nanofiber and enhanced photothermal immunotherapy (PTIT) for tumor suppression and prevention of recurrence using combined PSP@IR‐CF_3_ nanofibers and HSP70/AKT/mTOR axis inhibition.

## 2 Results

### 2.1 Preparation and Characterization of PSP@IR‐CF_3_

Using a solution‐based self‐assembly method, we successfully synthesized PSP@IR‐CF_3_ nanofibers. First, we chemically synthesized PSP and IR‐CF_3_ separately and confirmed their structures and purity for subsequent self‐assembly (Figures S1–S4 and Table S1). Scanning electron microscopy (SEM) analysis demonstrated that PSP and IR‐CF_3_ could assemble into nanofibers (Figure 2a). We also explored the effect of different amounts of IR‐CF_3_ on the co‐assembly process leading to fiber formation. SEM results showed that all groups formed fibrous nanomaterials regardless of the amount of IR‐CF_3_ added (Figure S5). Additionally, we used atomic force microscopy (AFM) to measure the thickness of the fibers in different groups (Figure S6). The results indicated that the thickness of the fibers increased with the addition of IR‐CF_3_, which may be due to the involvement of more IR‐CF_3_ during the co‐assembly process. Elemental analysis showed that fluorine (F) from IR‐CF_3_ was uniformly present across the fibers, suggesting that IR‐CF_3_ was not merely adsorbed onto PSP, but actively involved in the self‐assembly process (Figure 2b,c and Figure S7). The dynamic light scattering (DLS) results indicated that PSP@IR‐CF_3_ was well‐dispersed in phosphate‐buffered saline (PBS) and Dulbecco's modified Eagle's medium (DMEM) (10% fetal bovine serum), with an average hydrodynamic diameter of 489.74 ± 34.12 nm and 598.91 ± 27.19 nm, respectively (Figure 2d and Figure S8). Its zeta potential in PBS was ‐10.74 ± 1.43 mV (Figure 2e). Further stability testing revealed that PSP@IR‐CF_3_ dispersed in PBS can be stably stored at 4°C for 56 days without significant changes in particle size (Figure 2f). Subsequently, we tested the stability of the co‐assembly by incorporating fluorescein isothiocyanate (FITC) into the system. The results showed that less than 1% of IR‐CF_3_ was released, whereas over 70% of FITC was released after 24 h at 37°C and 100 rpm (Figure 2g). These findings suggest that a relatively stable co‐assembly structure is formed between IR‐CF_3_ and PSP, rather than simple incorporation. The circular dichroism (CD) results showed that the PSP@IR‐CF_3_ assembled into the β‐sheet (Figure 2h). Surface plasmon resonance (SPR) experiments demonstrated that, despite the presence of IR‐CF_3_, the nanofibers retained strong binding affinity (27.8 ± 2.2 nM) with the PD‐L1 protein, highlighting their potential for targeted therapy (Figure S9). UV–vis absorption spectra revealed notable absorption peaks in the near‐infrared region, particularly at 808 nm and 825 nm, indicating that the nanofibers are suitable for PTIT (Figure 2i). Additionally, molecular dynamics (MD) simulations were performed to further investigate the self‐assembly behavior of PSP and IR‐CF_3_. This dynamic process involved the movement of PSP and organic molecules, forming stable self‐assembled vesicles, which maintained their structure through dynamic equilibrium (Figure 2j–m; Figures S10 and S11). The interactions between PSP and IR‐CF_3_ molecules primarily comprised hydrophobic interactions, van der Waals forces, electrostatic interactions, and hydrogen bonds. Based on energy analysis of the binding kinetics process, during the interaction between the peptide and the ligand, the van der Waals energy was ‐222.509 kJ mol^−1^, and the electrostatic interaction energy was ‐277.396 kJ mol^−1^. These two forces constituted the main interactions, with a total energy of ‐384.650 kJ mol^−1^. The polypeptide molecules and the organic molecules were tightly bound through van der Waals forces. The halogen atoms in the organic molecules interacted with PHE5 and TYR4 through van der Waals forces and hydrophobic interactions. This close interaction facilitated the formation of the self‐assembled system. π‐π conjugation between two organic molecules stabilized the structure, forming a stable self‐assembled structure located within the polypeptide structure (Figure 2n and Table S2). The above results indicate a stable fibrous morphology, strong interactions with PD‐L1, and excellent photothermal properties of the self‐assembled nanofibers. The MD simulations further confirmed the stability and vesicle‐like structure of the assemblies, with both PSP and IR‐CF_3_ molecules contributing to the overall stability. These nanofibers show remarkable potential for applications in photothermal and immunotherapy.

**FIGURE 2:**
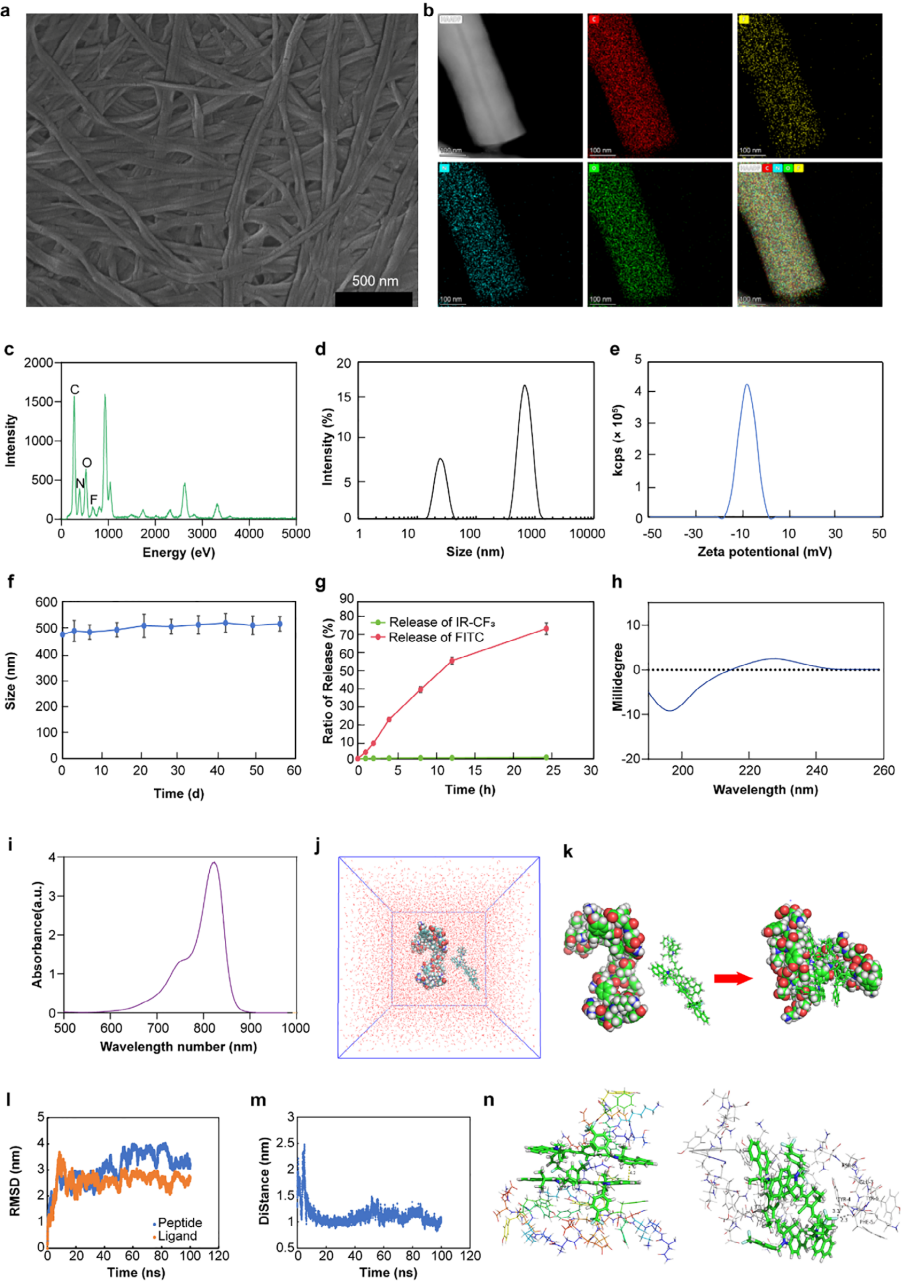
Characterization of PSP@IR‐CF_3_ nanofibers. (a) Scanning electron microscope (SEM) results of PSP@IR‐CF_3_. (b) Energy dispersive spectroscopy (EDS) mapping results of C, F, N, and O. (c) EDS spectrum results of PSP@IR‐CF_3_. (d) Dynamic light scattering (DLS) results of PSP@IR‐CF_3_ in phosphate‐buffered saline (PBS). (e) Zeta potential distribution of PSP@IR‐CF_3_ dispersed in PBS. (f) Changes in DLS particle size of PSP@IR‐CF_3_ dispersed and stored in PBS over 56 days. (g) Release curves of IR‐CF_3_ and fluorescein isothiocyanate (FITC) from PSP@IR‐CF_3_/FITC within 24 h at 37°C and 100 rpm. (h) Circular dichroism spectroscopy results of PSP@IR‐CF_3_. (i) Ultraviolet absorption spectroscopy results of PSP@IR‐CF_3_. (j) Initial system setup for the assembly process of IR‐CF_3_ with PSP. (k) Different self‐assembly states of PSP and IR‐CF_3_ at 0 ns and 100 ns. (l) Root mean square deviation results of PSP and IR‐CF_3_. (m) Distance changes between PSP and IR‐CF_3_ during assembly. (n) Force analysis results of the nanofibers.

### 2.2 PTIT Effects and Cellular Binding of PSP@IR‐CF_3_

To explore the PTIT characteristics of the nanofibers, they were laser‐irradiated at 808 nm under different conditions and their temperature changes were recorded using thermography. PSP@IR‐CF_3_ temperature varied depending on the nanofiber concentration, irradiation time, and laser power (Figure 3a–c and Figure S12). After 3 min of irradiation (1.0 W/cm^2^), the maximum temperatures of PSP@IR‐CF_3_ and IR‐CF_3_ (50 µg mL^−1^) increased by 22.79 ± 0.58°C and 25.64 ± 0.41°C, respectively, followed by a subsequent decline in temperature (Figure 3a,b). The temperature of PBS group remained relatively unchanged after irradiation. Compared with the control photothermal agent IR‐CF_3_, PSP@IR‐CF_3_ retained excellent and safe photothermal performance. As PSP@IR‐CF_3_ concentration increased (Figure 3c), the heating capability also improved. Additionally, with the increasing laser power, the temperature of PSP@IR‐CF_3_ also increased (Figure S12).

**FIGURE 3:**
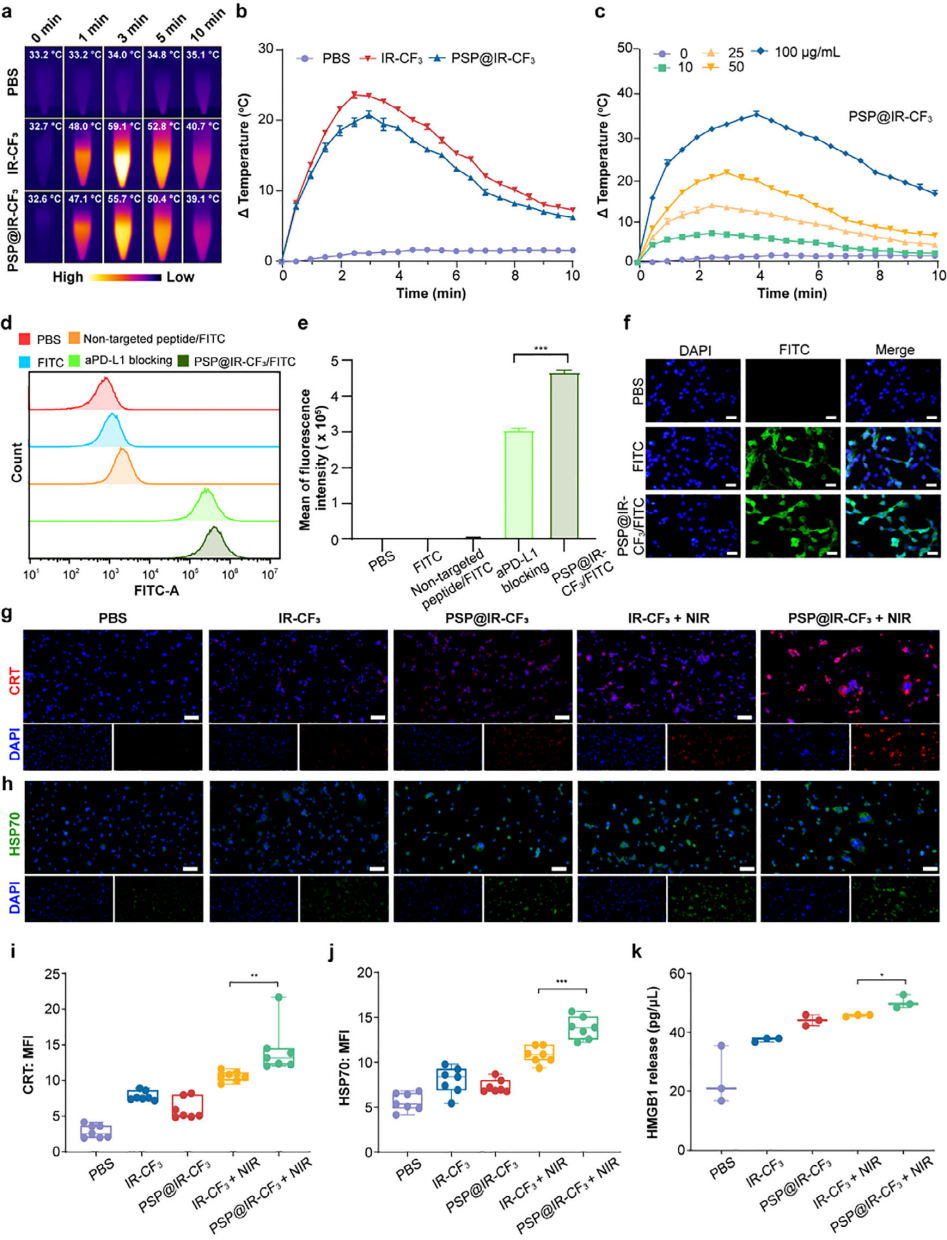
Photothermal immunotherapy (PTIT) effects and tumor targeting of PSP@IR‐CF_3_. (a) Temperature variations at different time points during NIR irradiation of PSP@IR‐CF_3_, IR‐CF_3_, and PBS control groups. The statistical analysis is presented in (b), where Δ Temperature = temperature at different time points—initial temperature (*n* = 3). (c) Temperature changes at different time points for varying concentrations of PSP@IR‐CF_3_ under NIR irradiation (*n* = 3). (d, e) Flow cytometry analysis showing the targeting specificity of PSP@IR‐CF_3_/FITC towards CT26‐Luc cells (*n* = 3). One‐way ANOVA followed by Tukey's multiple‐comparison test was used for statistical analysis. (f) Colocalization of PSP@IR‐CF_3_ with CT26‐Luc cells by immunofluorescence staining. Scale bars: 20 µm. Two‐way ANOVA was used for statistical analysis. (g, h) Immunofluorescence staining showing the expression of CRT and HSP70. Blue: DAPI (nuclei staining), red: CRT, green: HSP70. (i, j) Mean fluorescence intensity (MFI) of CRT and HSP70 immunofluorescence staining (*n* = 7). One‐way ANOVA followed by Tukey's multiple‐comparison test was used for statistical analysis. (k) Enzyme‐linked immunosorbent assay results showing the concentration of HMGB1 released by tumor cells into the culture medium (*n* = 3). Scale bars: 20 µm. One‐way ANOVA followed by Tukey's multiple‐comparison test was used for statistical analysis. The data are presented as means ± standard error of the mean (SEM). A value of *p* < 0.05 was considered statistically significant. Statistical significance is indicated as follows: *p* < 0.05 (*), *p* < 0.01 (**), and *p* < 0.001 (***).

We further examined the binding specificity of PSP@IR‐CF_3_ to CT26‐Luc cells in vitro. CT26‐Luc cells were incubated with PSP@IR‐CF_3_/FITC, PSP@IR‐CF_3_/FITC with aPD‐L1 blocking, non‐targeted peptide/FITC (Figure S13), free FITC, and PBS for 1 h, followed by flow cytometry to assess their targeting ability (Figure 3d,e). The mean fluorescence intensity values of CT26‐Luc cells were 465218.70 ± 9465.64 for the PSP@IR‐CF_3_/FITC group, 303709.00 ± 8716.50 for the aPD‐L1 blocking group, 2579.00 ± 128.37 for the non‐targeted peptide/FITC group, 1158.67 ± 38.75 for the FITC group, and 811.00 ± 19.92 for the PBS group. These results demonstrated that PSP@IR‐CF_3_ specifically targets and binds to PD‐L1 expressed in CT26‐Luc cells. Confocal microscopy results indicated that after co‐incubation of PSP@IR‐CF_3_/FITC with CT26‐Luc cells, a notable presence of green fluorescence was observed within the cells. This is because the docking of PSP with PD‐L1 causes the entire fiber structure to dissociate, releasing IR‐CF_3_ and FITC [33] (Figure 3f). These results substantiated the excellent photothermal properties and PD‐L1 targeting capability of PSP@IR‐CF_3_ in vitro, establishing a preliminary foundation for the in vivo application of nanofibers.

We further evaluated the therapeutic efficacy of PSP@IR‐CF_3_ in vitro. PTIT has recently been shown to induce ICD, which is characterized by high surface expression of calreticulin (CRT), heat shock protein 70 (HSP70), and the release of high mobility group box 1 (HMGB1) (Figure 3g–k). These signaling molecules are known as DAMPs and are associated with ICD. These effects were tested after treating CT26‐Luc cells with different nanofibers. The data showed that PSP@IR‐CF_3_ + NIR markedly increased the CRT expression of tumor cells compared to the IR‐CF_3_, PSP@IR‐CF_3_, and IR‐CF_3_ + NIR treatments (Figure 3g,i). The levels of HSP70 expression and the amount of HMGB1 secreted into the cell supernatant (Figure 3h,j–k) showed similar trends. Together with the flow cytometric analysis of CRT, HSP70, and HMGB1, these results confirmed the effective induction of ICD by PSP@IR‐CF_3_‐mediated PTIT (Figure S14). Importantly, the treated tumor cells effectively promoted DC maturation in the co‐culture system, indicating that the released DAMPs were functionally capable of stimulating antigen‐presenting cells (Figure S15). Collectively, these findings demonstrate that PSP@IR‐CF_3_‐mediated PTIT not only induces ICD but also facilitates the initiation of antitumor immune responses.

### 2.3 In Vivo Biodistribution and PTIT Capacity of PSP@IR‐CF_3_

We examined the *i*n vivo biodistribution and tumor‐targeting effects of PSP@IR‐CF_3_/IR‐808. CT26‐Luc tumor‐bearing mice were injected with PSP@IR‐CF_3_/IR‐808 or IR‐808 via the tail vein, and the biodistribution of nanofibers was analyzed with fluorescence molecular imaging (FMI) at different time points. Probe dosing was standardized across groups, with comparable pre‐injection fluorescence and qualities (Figure S16). PSP@IR‐CF_3_/IR‐808 was systemically distributed at 0.5 h post‐injection, and the fluorescence signal in the tumor region gradually increased over time (Figure 4a,b). The tumor fluorescence intensity of PSP@IR‐CF_3_/IR‐808 reached its maximum at 24 h post‐injection and remained detectable for more than 48 h. At this time point, PSP@IR‐CF_3_/IR‐808 exhibited approximately 1.6‐ and 2.5‐fold higher fluorescence intensity in tumors compared with the aPD‐L1 blocking and IR‐808 groups, respectively. In contrast, the fluorescence signals of the aPD‐L1 blocking and IR‐808 groups gradually decreased after reaching their maximum intensity. To further validate the in vivo FMI results, tumors and major organs were collected at 48 h post‐injection for ex vivo fluorescence imaging. Consistently, tumors from the PSP@IR‐CF_3_/IR‐808 group showed stronger fluorescence signals than those from the aPD‐L1 blocking and IR‐808 groups (Figure 4c,d). These results demonstrate enhanced tumor accumulation and retention of PSP@IR‐CF_3_/IR‐808, providing a basis for subsequent therapeutic studies.

**FIGURE 4:**
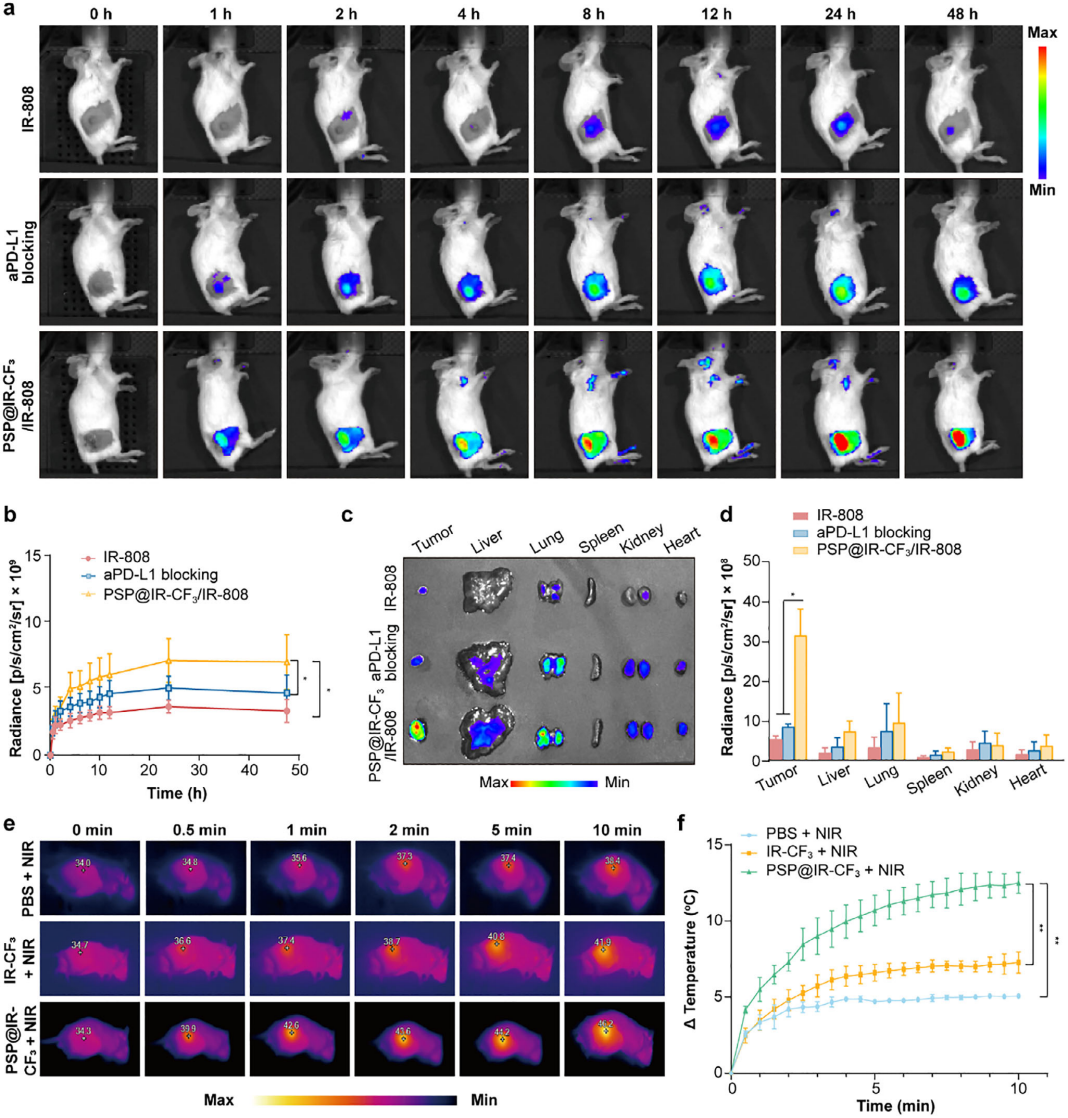
In vivo tumor targeting and photothermal immunotherapy (PTIT) effects of PSP@IR‐CF_3_. (a,b) Fluorescence intensity of PSP@IR‐CF_3_ biodistribution in CT26‐Luc tumor‐bearing mice (*n* = 3). Two‐way ANOVA was used for statistical analysis. (c,d) Ex vivo fluorescence imaging of organs from each group of mice at 48 h post‐treatment. Fluorescence intensity of each organ was quantified and statistically analyzed (*n* = 3). One‐way ANOVA followed by Tukey's multiple‐comparison test was used for statistical analysis. (e) Evaluation of the in vivo photothermal performance of PSP@IR‐CF_3_. Statistical results are detailed in (f), with Δ Temperature = temperature at different time points—initial temperature (*n* = 3). Two‐way ANOVA was used for statistical analysis. The data are presented as means ± SEM. A value of *p* < 0.05 was considered statistically significant. Statistical significance is indicated as follows: *p* < 0.05 (*), *p* < 0.01 (**), and *p* < 0.001 (***).

The CT26‐Luc tumor‐bearing mice were further treated with PBS, IR‐CF_3_, or PSP@IR‐CF_3_ for PTIT. After 24 h, the mice were exposed to 808 nm NIR irradiation (1.0 W/cm^2^), and the temperatures were measured with a thermal imaging device (Figure 4e). For the PSP@IR‐CF_3_ + NIR group, the tumor site temperature rose by 10°C within 5 min, reaching a peak of approximately 46.2°C after 10 min (Figure 4f). However, no significant temperature increase was observed in the IR‐CF_3_ + NIR and PBS + NIR groups. These results confirm the in vivo PTIT effects of PSP@IR‐CF_3_, indicating that the temperature rise was attributed to improved tumor targeting and increased accumulation of PSP@IR‐CF_3_ at tumor sites.

### 2.4 In Vivo PTIT of PSP@IR‐CF_3_

We studied the anti‐tumor effects of PSP@IR‐CF_3_ in CT26‐Luc tumor‐bearing mice. CT26‐Luc tumor‐bearing mice were selected because they are the commonly used models in studies of immunotherapy and photothermal therapies, including photoimmunotherapy. They exhibit high PD‐L1 expression and are considered an immunologically “cold” tumor type [40, 41, 42]. The treatment scheme is shown in Figure 5a. Representative images of the mice at different time points following treatment are presented in Figure 5b. The therapeutic response was assessed by monitoring the tumor volume and weight (Figure 5c,d and Figure S17). Tumor growth was not appreciably inhibited in the PBS, IR‐CF_3_, and PBS + NIR groups (Figure 5e–g). Mice treated with IR‐CF_3_ + NIR (Figure 5h), aPD‐L1 (Figure 5i), or PSP@IR‐CF_3_ (Figure 5j) showed certain anti‐tumor efficacy. The tumor‐suppressive effect of IR‐CF_3_ + NIR primarily resulted from its photothermal effect. Tumor growth was reduced, but not fully eradicated, in the aPD‐L1 and PSP@IR‐CF3 groups, indicating that neither NIR nor PSP@IR‐CF_3_ could sufficiently inhibit tumor growth independently. However, PSP@IR‐CF_3_ + NIR markedly inhibited tumor growth (Figure 5k) and significantly extended the survival time (Figure 5l). Additionally, the body weights of mice in the seven groups remained stable throughout treatment (Figure S18). The levels of biomarkers such as alanine aminotransferase (ALT) and aspartate aminotransferase (AST) remained within the safety thresholds (Figure S19). Hematoxylin and eosin (H&E) staining of major organs showed no histopathological abnormalities (Figure S20). These findings demonstrated the biosafety and biocompatibility of this treatment. Collectively, these results suggest that PSP@IR‐CF_3_ effectively and safely suppresses tumor growth.

**FIGURE 5:**
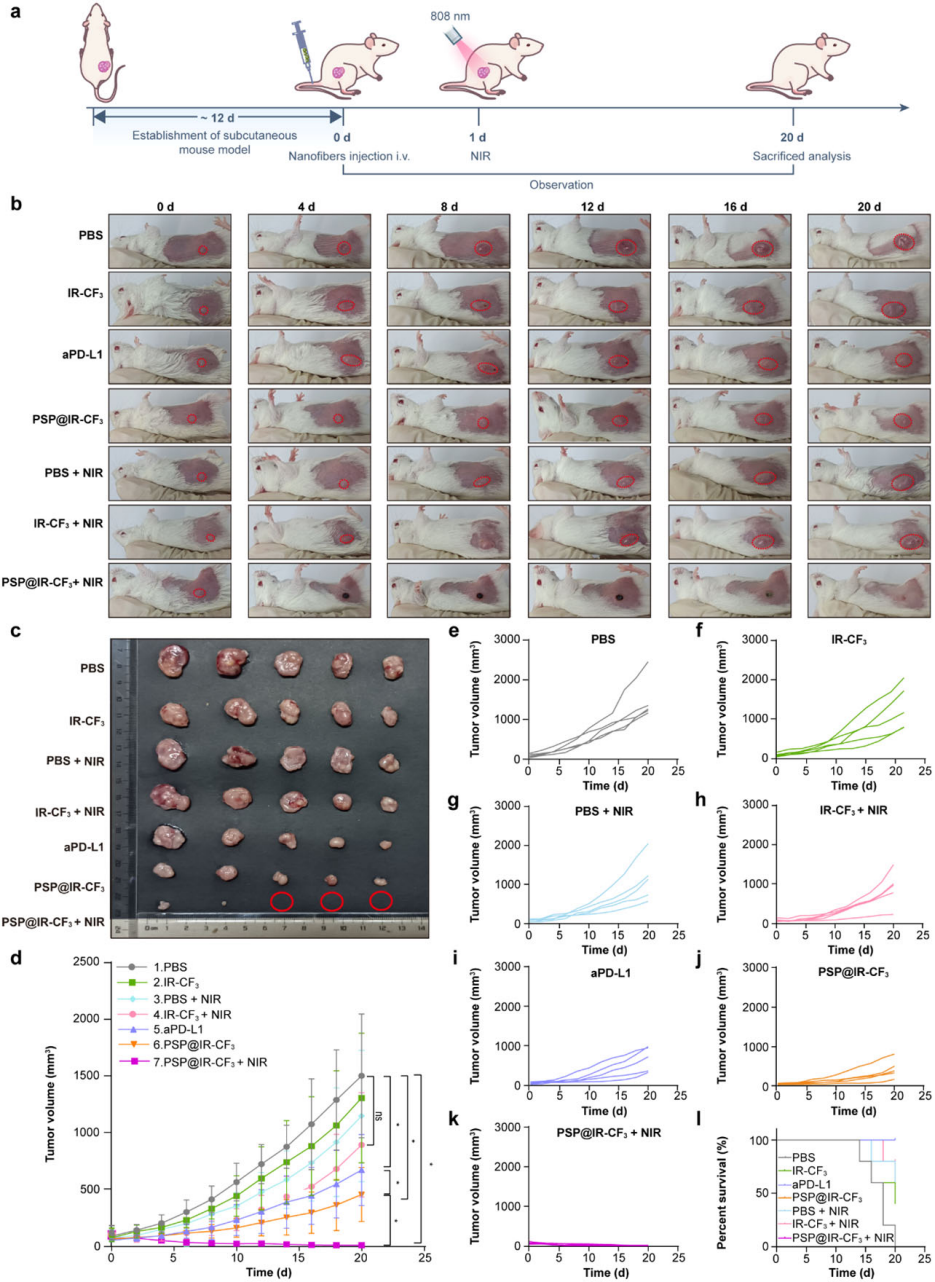
In vivo PTIT of PSP@IR‐CF_3_. (a) Schematic illustration of the in vivo PSP@IR‐CF_3_ PTIT in CT26‐Luc tumor‐bearing mice. (b) Representative images of mice at different time points with different treatments. (c) Ex vivo images of excised tumors from the treated mice. (d) Tumor volumes of different treatment groups (*n* = 5). Two‐way ANOVA was used for statistical analysis. (e–k) Tumor volume changes in individual mice during the treatments (*n* = 5). (l) Survival analysis of different treatment groups (*n* = 5). The data are presented as means ± SEM. A value of *p* < 0.05 was considered statistically significant. Statistical significance is indicated as follows: *p* < 0.05 (*), *p* < 0.01 (**), and *p* < 0.001 (***).

To demonstrate the general therapeutic application of PSP@IR‐CF_3_, we extended our investigation to 4T1‐Luc tumor‐bearing mice, which are known for their poor immunogenicity and low PD‐L1 expression. Compared to the CT26‐Luc tumor‐bearing mice, which exhibit relatively high levels of PD‐L1, 4T1‐Luc tumors provide a more immunologically challenging setting for evaluating PD‐L1‐targeted therapies. Our results showed that PSP@IR‐CF_3_ + NIR treatment resulted in appreciable tumor growth inhibition in the 4T1‐Luc tumor‐bearing mice as shown in Figure S21. The tumor suppression observed above suggests the potential applicability of PSP@IR‐CF_3_ across different tumor models.

### 2.5 PTIT with PSP@IR‐CF_3_ Induces ICD but Mitigates the Cytokine Storm

Furthermore, we examined the ability of PTIT with PSP@IR‐CF_3_ to induce ICD in vivo. Initially, we observed that PSP@IR‐CF_3_ + NIR induced a marked increase in cleaved caspase‐3 expression within tumor tissues, indicating the induction of programmed cell death in tumors (Figure 6a and Figure S22). Subsequently, we assessed the classical markers of ICD by immunofluorescence staining of tumors from various treatment groups, revealing a significant increase in cell‐surface CRT expression and extracellular release of HMGB1 in the PSP@IR‐CF_3_ + NIR group. In contrast, minimal expression was observed in other groups with CT26‐Luc tumors (Figure 6a). A similar phenomenon was observed in 4T1‐Luc tumors (Figure S23).

**FIGURE 6:**
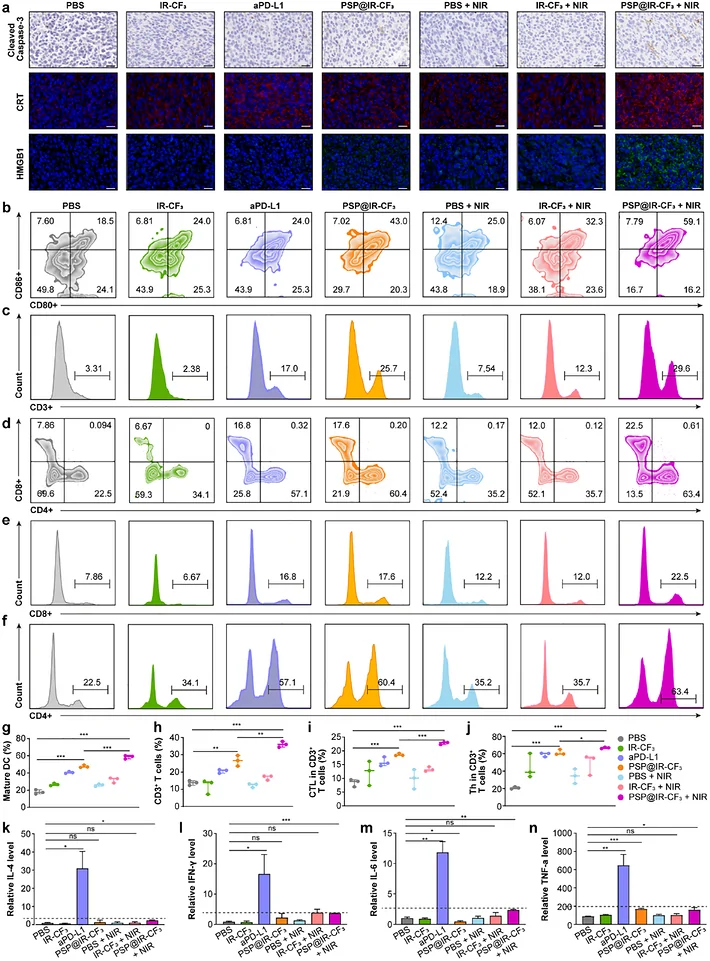
PTIT of PSP@IR‐CF_3_ induces apoptosis and ICD in vivo. (a) Immunohistochemical and immunofluorescence staining were used to detect the apoptotic marker cleaved caspase‐3 and ICD markers CRT and HMGB1. (b) CD80 and CD86 expression on the surface of dendritic cells (DCs). (c) CD3 expression on the surface of T cells. (d) Populations of CD8^+^ and CD4^+^ T cells. (e) Populations of CD3^+^CD8^+^ T cells. (f) Populations of CD3^+^CD4^+^ T cells. (g) Proportion of mature DCs (*n* = 3). (h) Proportion of CD3^+^ T cells (*n* = 3). (i) Proportion of cytotoxic T lymphocyte (CTL) cells among CD3^+^ T cells (*n* = 3). (j) Proportion of T helper (Th) cells among CD3^+^ T cells (*n* = 3). (k–n) Serum concentrations of cytokines IL‐4 (k), IFN‐γ (l), IL‐6 (m), and TNF‐α (n) (*n* = 3). Scale bars: 20 µm. One‐way ANOVA followed by Tukey's multiple‐comparison test was used for statistical analysis. The data are presented as means ± SEM. A value of *p* < 0.05 was considered statistically significant. Statistical significance is indicated as follows: *p* < 0.05 (*), *p* < 0.01 (**), and *p* < 0.001 (***).

DCs are antigen‐presenting cells crucial for initiating and regulating both innate and adaptive immunity. We therefore examined the impact of nanofiber‐induced ICD on the maturation of bone marrow‐derived DCs (BMDCs) in CT26‐Luc tumor‐bearing mice. After treatment, we collected and analyzed the spleens and tumors. The PSP@IR‐CF_3_ + NIR group had a significantly higher percentage of mature DCs compared to the other groups (Figure 6b,g and Figure S24). These data suggest that PSP@IR‐CF_3_ + NIR therapy effectively induces ICD and promotes DC maturation, contributing to enhanced antitumor immune activation. As shown in Figure 6c–f and h–j, the number of CD45^+^CD3^+^ T cells, including CD3^+^CD8^+^ and CD3^+^CD4^+^ T cells in splenocytes, was significantly enhanced in the PSP@IR‐CF_3_ + NIR group. Compared to the PBS group, the percentages of CD45^+^CD3^+^, CD3^+^CD8^+^, and CD3^+^CD4^+^ T cells in this group increased by approximately 8.9‐, 27.3‐, and 25.2‐fold, respectively (Figure 6h–j). Moreover, further evaluation of CD8^+^ T‐cell function revealed that the proportions of IFN‐γ^+^ and Granzyme B^+^ cells among CD8^+^ T cells were significantly increased after PSP@IR‐CF_3_ + NIR treatment (Figure S25), indicating enhanced cytokine production and cytotoxic effector function of CD8^+^ T cells. Flow cytometric analyses demonstrated that PTIT alone or ICB alone induced limited immune activation, whereas the combination of PTIT with PSP@IR‐CF_3_ promoted DC maturation and functional T‐cell activation, contributing to enhanced antitumor immunity. Comparable results were obtained in the 4T1‐Luc tumor model (Figure S26).

Furthermore, PTIT of PSP@IR‐CF_3_ significantly elevated the levels of cytokines IL‐4, TNF‐α, IFN‐γ, and IL‐6 in vivo. Compared to the control group, the PSP@IR‐CF_3_ + NIR group exhibited a 2.38‐fold increase in IL‐4 levels (2.38 ± 0.25 vs. 1.00 ± 0.32), a 4.35‐fold increase in IFN‐γ levels (3.66 ± 0.06 vs. 0.84 ± 0.22), a 2.35‐fold increase in IL‐6 levels (2.35 ± 0.11 vs. 1.00 ± 0.19), and a 1.80‐fold increase in TNF‐α levels (159.68 ± 23.56 vs. 88.77 ± 0.99). Furthermore, the aPD‐L1 group showed substantially elevated cytokine levels: IL‐4 increased by 30.95‐fold (30.95 ± 9.39 vs. 1.00 ± 0.32), IFN‐γ increased by 19.81‐fold (16.64 ± 8.05 vs. 0.84 ± 0.22), IL‐6 increased by 11.83‐fold (11.83 ± 2.12 vs. 1.00 ± 0.19), and TNF‐α increased by 7.32‐fold (649.39 ± 116.81 vs. 88.77 ± 0.99). Excessive elevation of inflammatory cytokines, particularly IL‐6, has been associated with cytokine release syndrome and systemic immune activation. Therefore, these cytokine profiles were evaluated to assess potential systemic immune activation and treatment safety (Figure 6k–n).

### 2.6 Effects of Combination Therapy on Systemic Antitumor Immune Responses

To assess the anti‐tumor efficacy of combination therapy on systemic immunological responses, mouse models with bilateral tumors were used. Once the primary tumor reached approximately 50 mm^3^ in volume, PSP@IR‐CF_3_ was intravenously administered via the tail vein. At 24 h post‐injection, the primary tumors were exposed to NIR irradiation (Figure 7a).

**FIGURE 7:**
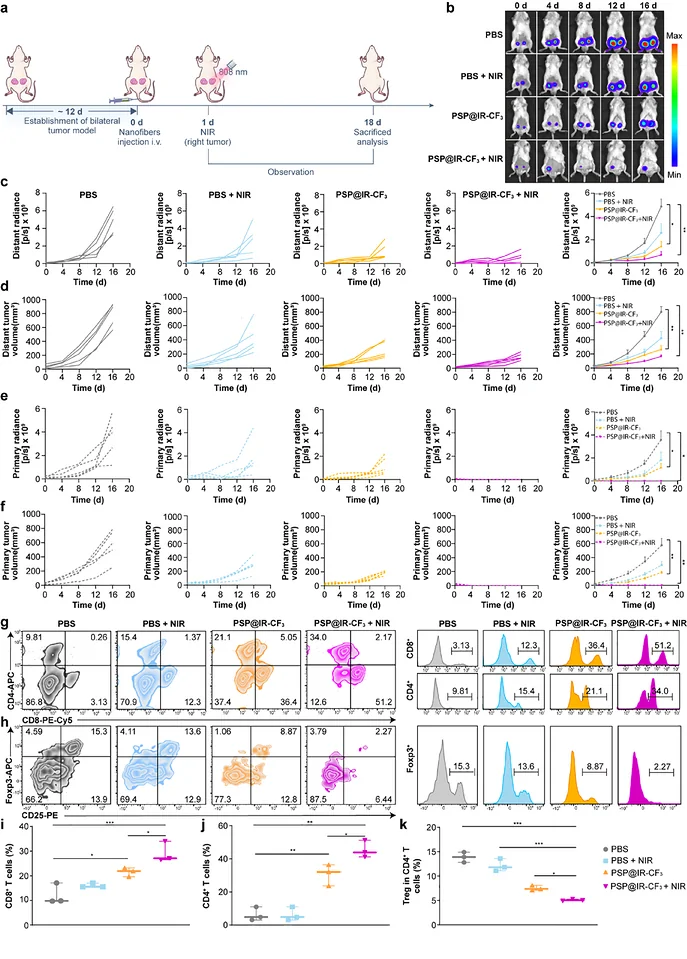
Effects of PTIT on distant tumors and the immunological response. (a) Schematic illustration of the in vivo PSP@IR‐CF_3_ PTIT protocol in the bilateral tumor mice. (b) Representative bioluminescence imaging (BLI) of the bilateral tumor mice at different time points after different treatments. (c–f) Statistical analysis of tumor volume and BLI signal intensities in different treatment groups (*n* = 5). Two‐way ANOVA was used for statistical analysis. (g–k) Immunomodulatory effects of PSP@IR‐CF_3_ + NIR. Statistical analysis of CD3^+^CD8^+^ CTLs (g,i), CD3^+^CD4^+^ Th cells (g,j), and CD4^+^CD25^+^Foxp3^+^ Tregs (h,k) in different treatment groups (*n* = 3). One‐way ANOVA followed by Tukey's multiple‐comparison test was used for statistical analysis. The data are presented as means ± SEM. A value of *p* < 0.05 was considered statistically significant. Statistical significance is indicated as follows: *p* < 0.05 (*), *p* < 0.01 (**), and *p* < 0.001 (***).

The results demonstrated that PSP@IR‐CF_3_ + NIR treatment significantly inhibited tumor growth at both the primary and distant sites, compared with the control group (Figure 7b–f). In the PSP@IR‐CF_3_ + NIR group, substantial therapeutic effects were observed on primary tumors, with significant inhibition. Distant tumors also exhibited a notable therapeutic response, indicating that PSP@IR‐CF_3_ + NIR elicited a systemic antitumor effect. The inhibitory effect at distant tumors began to emerge 4 d post‐treatment, suggesting that immune‐mediated effects at distant tumors may require additional time to develop compared with the direct photothermal effects observed at primary tumors. Furthermore, the group treated with PSP@IR‐CF_3_ alone showed moderate tumor inhibition compared with the control group, suggesting that PSP@IR‐CF_3_ alone possesses antitumor activity, potentially due to its ICB capabilities. Based on this hypothesis, we analyzed immune cells within the TME, focusing on immune T cells.

The results showed significant immune activation of the TME in the PSP@IR‐CF_3_ + NIR group. The proportions of CD3^+^CD8^+^ and CD3^+^CD4^+^ T cells were markedly higher than those in the control group, with CD3^+^CD8^+^ T cells increasing approximately 16‐fold and CD3^+^CD4^+^ T cells increasing about 3.5‐fold (Figure 7g,i–j). In contrast, the proportion of CD4^+^CD25^+^Foxp3^+^ regulatory T cells (Tregs) decreased by approximately 6.7‐fold (Figure 7h,k), indicating that PSP@IR‐CF_3_ + NIR activated the immune microenvironment and reduced immunosuppressive features. This comprehensive evaluation highlighted the functionality of PSP@IR‐CF_3_ + NIR therapy in targeting both primary and distant tumors. PSP@IR‐CF_3_‐mediated PTIT achieved local tumor control and promoted systemic antitumor immune responses in a bilateral tumor model, highlighting its potential for future applications in metastatic cancer treatment.

### 2.7 Activation of the HSP70/AKT/mTOR Axis Following NIR‐PTIT Contributes to Tumor Recurrence

Although PTIT has shown effective therapeutic outcomes, tumor recurrence remains a significant challenge, with some tumors exhibiting resistance to PTIT (Figure S27). To further understand the mechanism of adaptive resistance, we conducted transcriptomic sequencing of recurrent tumors from the PSP@IR‐CF_3_ + NIR group as well as tumors from the PSP@IR‐CF_3_ and PBS control groups. Our analysis revealed that 20,700 genes were detected in all groups (Figure S28). Volcano plots indicated that, in the PSP@IR‐CF_3_ + NIR group, compared to the PBS group, 2,657 genes were upregulated, whereas 1,729 genes were downregulated (Figure 8a). Additionally, compared with the PSP@IR‐CF_3_ group, the PSP@IR‐CF_3_ + NIR group had 2,888 upregulated genes and 2,099 downregulated genes (Figure S29). To identify the pathways potentially associated with tumor recurrence, we performed a Kyoto Encyclopedia of Genes and Genomes (KEGG) pathway analysis, comparing the PSP@IR‐CF_3_ + NIR group with either PBS or PSP@IR‐CF_3_ groups. The analysis revealed significant upregulation of the PI3K/AKT/mTOR pathway (Figure 8b,c and Figure S30). The observed activation of the PI3K/AKT/mTOR pathway suggests a potential survival mechanism that may contribute to tumor recurrence. In contrast, pathway analysis of the downregulated genes (Figure 8d and Figure S31) revealed strong suppression of oxidative phosphorylation, steroid metabolism, DNA replication, ribosome biogenesis, and cell cycle progression. These findings suggest that PSP@IR‐CF_3_ + NIR effectively impairs tumor metabolism and induces cell death.

**FIGURE 8:**
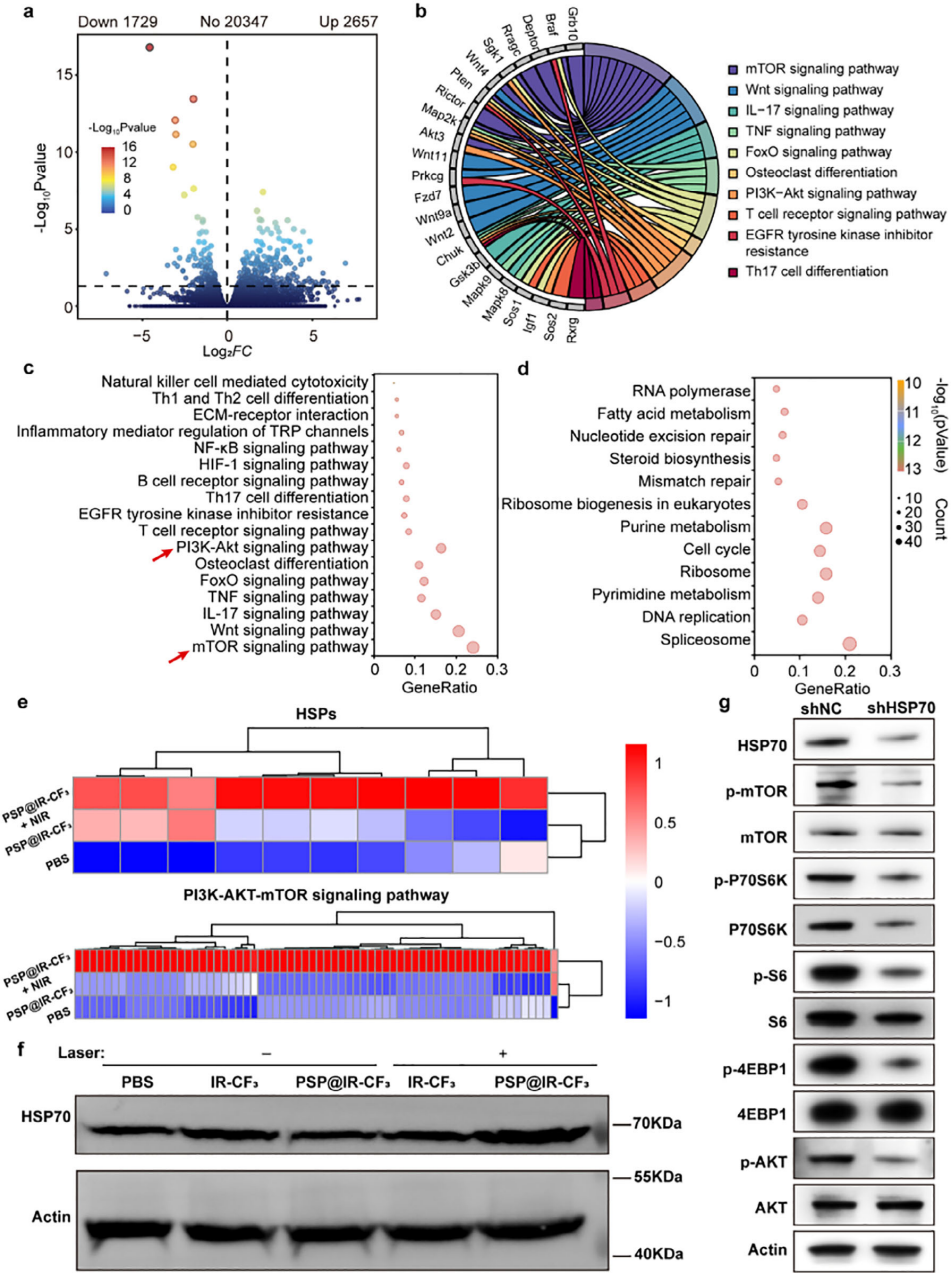
The mechanism analysis of tumor recurrence after PTIT of PSP@IR‐CF_3_ by RNA sequencing. (a) The volcano plot of differentially expressed genes between the PSP@IR‐CF_3_ + NIR and PBS groups. (b,c) Kyoto Encyclopedia of Genes and Genomes (KEGG) pathway enrichment of upregulated genes in PSP@IR‐CF_3_ + NIR and PBS groups (red arrows represent HSP70/AKT/mTOR relative pathways). (d) Genome‐related pathway enrichment of downregulated genes in PSP@IR‐CF_3_ + NIR and PBS groups. (e) Heatmap of HSPs and PI3K/AKT/mTOR expression levels in PSP@IR‐CF_3_ + NIR, PSP@IR‐CF_3_, and PBS groups. (f) Western blotting of HSP70 (*n* = 3). (g) Western blotting of HSP70 and PI3K/AKT/mTOR (*n* = 3).

Furthermore, PSP@IR‐CF_3_ alone induced an immune activation‐associated transcriptional profile. Compared with PBS treatment, PSP@IR‐CF_3_ treatment resulted in upregulation of immune‐related pathways, including T cell receptor signaling, antigen presentation‐associated pathways, and natural killer cell‐mediated cytotoxicity (Figure S32). Conversely, genes involved in oxidative phosphorylation and steroid metabolism were downregulated, suggesting metabolic alterations associated with immune activation and reduced tumor metabolic activity (Figure S33). These findings suggest that although PSP@IR‐CF_3_ initiates immune stimulation and metabolic suppression, the addition of NIR irradiation further strengthens these effects.

Previous research has indicated that HSP70 activates the mTOR pathway [43]. Given that PTIT acts as a stressor that induces the expression of HSPs, including HSP70, we hypothesized that PTIT‐induced HSP70 expression may contribute to activation of the PI3K/AKT/mTOR pathway (Figure 8e). To investigate this possibility, we measured HSP70 expression in tumor tissues following PTIT (Figure 8f), observing a significant increase in HSP70 protein levels. Subsequently, we knocked down HSP70 in CT26‐Luc cells, examined the PI3K/AKT/mTOR pathway, and observed a reduction in its activation (Figure 8g; Figures S34 and S35). To further evaluate the functional significance of this pathway, colony formation assays were performed following PTIT. Consistent with the proposed mechanism, treatment with VER‐155008 or Rapamycin reduced colony formation and impaired clonogenic recovery of residual tumor cells after PTIT (Figure S36). Collectively, these findings suggest that HSP70 upregulation is associated with activation of the PI3K/AKT/mTOR pathway and may contribute to post‐treatment tumor survival and recurrence.

### 2.8 PTIT Combined with mTOR Inhibition Reduces Tumor Recurrence in Preclinical Models

NIR‐PTIT can activate the immune response; however, it may also trigger cellular stress, elevate HSP70 levels, and activate the PI3K/AKT/mTOR signaling pathway, which may contribute to adaptive resistance and tumor recurrence. To address this issue, the specific mTOR inhibitor and FDA‐approved drug rapamycin was administered immediately after NIR irradiation as a post‐PTIT adjunctive intervention to suppress PI3K/AKT/mTOR pathway activity, thereby enhancing PTIT efficacy and reducing recurrence rates (Figure 9a). In the subcutaneous mouse CT26‐Luc tumor models, 6 out of 20 mice experienced recurrence, yielding a recurrence rate of 30% in the PSP@IR‐CF_3_ + NIR group. In contrast, no recurrence was observed in the 20 mice treated with PSP@IR‐CF_3_ + NIR combined with rapamycin (Figures S37–S39). Likewise, no recurrent tumors were detected in mice receiving PTIT combined with the HSP70 inhibitor VER‐155008 throughout the observation period (Figures S40 and S41). To better replicate clinical conditions, patient‐derived clinical samples from patients with breast cancer were used to establish an in situ murine patient‐derived xenograft (PDX) model, demonstrating notable therapeutic outcomes and exceptionally high safety in the combined treatment group (Figure 9b,c). Ex vivo tumor images from the PBS, PSP@IR‐CF_3_, PSP@IR‐CF_3_ + NIR, and PSP@IR‐CF_3_ + NIR + Rapa groups revealed marked differences in tumor burden (Figure 9d,e). Notably, the PSP@IR‐CF_3_ + NIR + Rapa group achieved a 100% cure rate (5/5 mice), whereas PSP@IR‐CF_3_ + NIR showed a partial response with a 60% cure rate (3/5). PSP@IR‐CF_3_ monotherapy also exhibited certain tumor growth inhibition [TGI = (1− treatment group average tumor weight / control group average tumor weight) ×100%], with the average tumor weight reduced by 36.4% compared to the PBS group, supporting its antitumor activity (Figure 9e and Table S3). Importantly, to evaluate potential systemic toxicity, we performed both histopathological and serum biochemical analyses. Major organs including the heart, liver, spleen, lungs, and kidneys were collected from treated PDX mice and subjected to H&E staining, which revealed no observable histopathological abnormalities (Figure S42). In parallel, serum biochemical parameters such as AST, ALT, blood urea nitrogen (BUN), and creatinine (CREA) remained within normal physiological ranges, indicating no detectable hepatotoxicity or nephrotoxicity (Figure S43). These findings support the favorable systemic tolerability of the combination therapy in this model.

**FIGURE 9:**
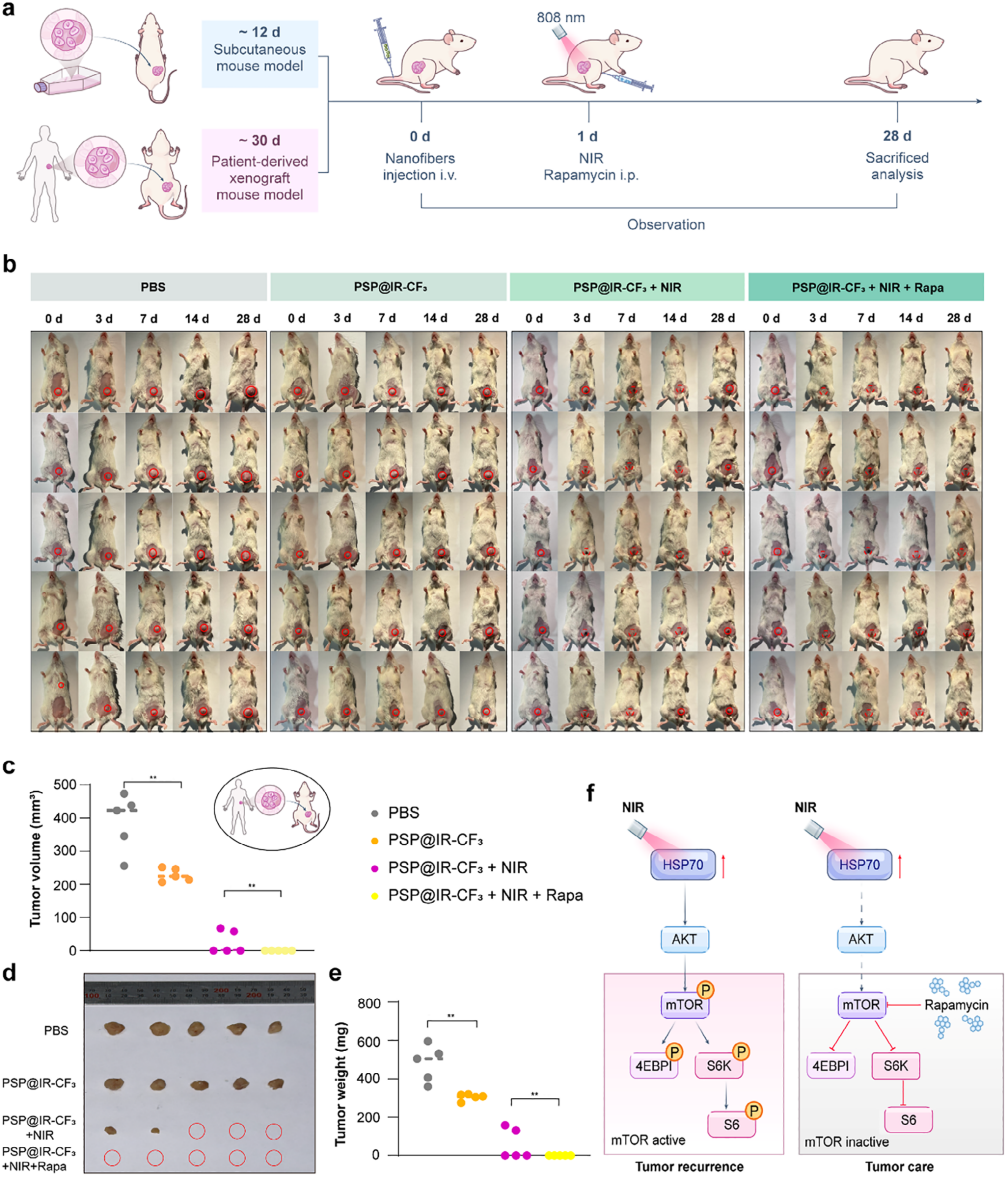
Therapeutic efficacy of the combination treatment with PSP@IR‐CF_3_ + NIR and rapamycin. (a) Schematic illustration of PSP@IR‐CF_3_ + NIR and rapamycin treatment protocol in the subcutaneous CT26‐Luc and patient‐derived xenograft (PDX) mouse model. (b) Representative images of PDX mouse models at different time points after different treatments (*n* = 5). (c) Tumor volume measurements in PDX mouse models on Day 28 (*n* = 5). (d) Ex vivo images of excised tumors from the treated PDX mouse models (*n* = 5). (e) Tumor weight measurements in PDX mouse models (*n* = 5). (f) Mechanism of the combined therapy in inhibiting tumor growth and recurrence. Rapa, rapamycin.

In summary, these results support the enhanced efficacy of the combination treatment, with reduced tumor recurrence and limited treatment‐associated toxicity in preclinical models. Collectively, our findings suggest that PTIT‐induced HSP70 upregulation is associated with activation of the PI3K/AKT/mTOR pathway and may contribute to post‐treatment tumor survival and recurrence. Consistent with this observation, rapamycin treatment reduced tumor recurrence and improved therapeutic outcomes, further supporting the involvement of mTOR signaling in post‐treatment tumor survival. (Figure 9f).

## 3 Discussion

Clinical cancer immunotherapy faces several challenges, including low immune response rates and limited efficacy, particularly in treating “cold” tumors [44, 45]. To address these issues, we developed a novel carrier‐free, self‐assembled PTIT nanodelivery system, PSP@IR‐CF_3_, which integrates tumor‐targeted delivery, immune checkpoint blockade, and photothermal function while minimizing toxicity. The PD‐L1‐targeting immune‐peptide, PSP, specifically binds to PD‐L1 expressed on tumor cell surfaces, inhibiting tumor immune evasion by blocking the PD‐1/PD‐L1 pathway. Meanwhile, IR‐CF_3_, acting as a photothermal response component, converts light energy into heat under NIR irradiation, activating the TME and enhancing immune responses. Unlike traditional external carrier‐based delivery systems, the self‐assembled, carrier‐free PSP@IR‐CF_3_ nanofibers avoid issues related to nanocarrier safety, uncontrolled drug release, and inefficient targeting. This self‐assembly structure improves drug biodistribution and targeting to the tumor site and extends the drug's half‐life and circulation time, reducing systemic exposure and minimizing side effects. Furthermore, PSP@IR‐CF_3_ enables multifaceted therapeutic effects with a single dose, increasing the combined immunotherapeutic effects with reduced toxicity, simplified treatment, and improved clinical feasibility.

Regarding therapeutic efficacy, PSP@IR‐CF_3_ plus NIR outperforms traditional PD‐L1 antibody immunotherapy. The mild PTIT activates the immune microenvironment, enhances tumor tissue permeability, reduces damage to normal tissues, and promotes synergistic effects between immunotherapy and photothermal therapy [46, 47]. PSP@IR‐CF_3_ plus NIR effectively induces the ICD effect, which directly damages tumor cells and releases a range of immune‐stimulatory molecules, such as HMGB1. These released molecules promote the activation of DCs and facilitate antigen‐presentation‐related immune responses, which are associated with enhanced CD8^+^ T‐cell activation and cytotoxic function, thereby improving antitumor immunity [46, 48, 49, 50]. Building on this evidence of immune activation, future studies will further characterize antigen‐specific T‐cell priming and long‐lasting immunological memory using antigen‐specific T‐cell assays and tumor rechallenge models. More importantly, unlike traditional immunotherapies, PSP@IR‐CF_3_ shows a favorable safety profile with a low risk of cytokine release syndrome, primarily due to the spatiotemporal precision of PTIT, which induces ICD specifically within the irradiated tumor microenvironment. This localized ICD triggers the release of tumor‐associated antigens and DAMPs, recruiting and activating antigen‐presenting cells such as DCs. This immune activation is well‐regulated, avoiding widespread inflammation. In our study, PSP@IR‐CF_3_ combined with NIR enhanced local immune cell infiltration and induced strong CD8^+^ T cell responses, while circulating levels of IL‐6, IFN‐γ, and TNF‐α remained within physiological ranges, indicating effective immunity without systemic overstimulation [51, 52]. Notably, PSP@IR‐CF_3_ + NIR exhibited comparable therapeutic efficacy across tumor models with different basal PD‐L1 expression levels, suggesting that PD‐L1‐directed targeting is not the sole contributor to the therapeutic response. PD‐L1‐independent mechanisms, including passive tumor accumulation, photothermal effects, and PTIT‐induced ICD, also contribute to the overall antitumor efficacy, while photothermal treatment‐induced dynamic PD‐L1 upregulation may further support subsequent checkpoint blockade [20].

Despite these promising results, PTIT may still encounter adaptive resistance in recurrent tumors, potentially limiting its long‐term therapeutic efficacy. Our transcriptomic analysis identified activation of the HSP70/AKT/mTOR axis as a potential mediator of this resistance. To investigate the therapeutic relevance of this finding, we further combined PSP@IR‐CF_3_ with rapamycin, a selective mTOR pathway inhibitor [53, 54]. Notably, rapamycin significantly suppressed clonogenic recovery in vitro and reduced tumor recurrence in vivo, supporting a role of mTOR signaling in post‐treatment tumor survival. This combination strategy improved long‐term therapeutic efficacy and provides a rationale for further mechanistic investigation and preclinical evaluation of mTOR‐targeted combination therapies. Given the important roles of mTOR signaling in immune regulation, the potential immunomodulatory effects of rapamycin should also be considered. Although our results support its role in suppressing tumor‐intrinsic adaptive resistance, further studies are warranted to clarify its effects on immune cell metabolism and immune regulation in the context of PTIT. Moreover, adaptive resistance is likely governed by a complex signaling network, and the precise mechanisms underlying this process remain to be fully elucidated. Compensatory activation of alternative pathways, including MAPK/ERK, NF‐κB, and STAT3 signaling, may also contribute to tumor survival and therapeutic resistance. Future studies are therefore needed to systematically dissect these signaling networks and adaptive mechanisms following PTIT treatment.

Nevertheless, several limitations remain. First, the penetration depth of NIR light is intrinsically restricted (∼5–10 mm), which may hinder applications in deeply seated tumors [55]. Clinical strategies such as optical fiber‐guided illumination, currently being evaluated in ongoing PIT trials (e.g., ASP‐1929), may aid in addressing this challenge [56]. Second, although PSP@IR‐CF_3_ demonstrated consistent anti‐tumor effects in CT26‐Luc and 4T1‐Luc models, broader validation in additional tumor types, particularly deeply immunosuppressed “cold” tumors, is warranted. Third, although our peptide‐dye self‐assembly is well‐defined and reproducible, further studies on pharmacokinetics, biodistribution, and scalable Good Manufacturing Practice‐grade synthesis are essential to support future clinical translation.

In summary, PSP@IR‐CF_3_ offers a great opportunity for clinical translation due to its simple self‐assembled nanofiber structure, which significantly enhances therapeutic efficacy while reducing systemic toxicity. Its ability to deliver multi‐faceted therapeutic effects with a single dose simplifies treatment regimens, improving patient compliance and efficacy. The combination with rapamycin, an FDA‐approved mTOR inhibitor, further supports the clinical relevance of targeting tumor adaptive resistance through mTOR pathway modulation. This approach enhances therapeutic outcomes and may help overcome treatment resistance, providing a promising and effective strategy for treating “cold tumors” and advancing cancer immunotherapy.

## 4 Conclusions

In summary, we developed a carrier‐free, self‐assembled PSP@IR‐CF_3_ nanofiber platform that integrates PD‐L1‐targeted immune modulation and PTIT. This multifunctional nanoplatform effectively induced ICD, promoted DC activation, and enhanced T‐cell‐mediated antitumor immune responses, resulting in improved therapeutic efficacy across multiple tumor models with favorable biosafety. Furthermore, we identified PTIT‐induced HSP70/AKT/mTOR signaling activation as a potential contributor to adaptive tumor survival, and demonstrated that mTOR inhibition with rapamycin reduced tumor recurrence. Collectively, this study presents a peptide‐guided, carrier‐free nanotherapeutic strategy that combines immune activation and resistance intervention, providing a potential approach for improving therapeutic efficacy.

## 5 Experimental Section

### 5.1 Synthesis of IR‐CF_3_

IR‐CF_3_ was synthesized through a two‐step reaction. Specifically, the 1,1,2‐Trimethyl‐^1^H‐benzo[e]indole (9.8 g) and 4‐(Trifluoromethyl) Benzyl Bromide (12.3 g) were dissolved in 40 mL o‐dichlorobenzene and stirred under 120°C for 30 min. The solid crude product was then filtered and recrystallized in methanol. The purified product 1 (10.06 g, 62%) was dried under vacuum. 80 mL DMF/dichloromethane mixture (1/1, v/v) was added dropwise into a solution of phosphoryl chloride (37 mL) and anhydrous dichloromethane (35 mL) under stirring at 0°C. Afterwards, 10 g cyclohexanone was added dropwise, and the solution was then heated and refluxed for 3 h. The mixture was poured onto ice, yielding a solid product which was collected by filtration and washed with iced diethyl ether. The resulting yellow product (2‐chloro‐3‐(hydroxymethylidene) cyclohexene‐1‐carbaldehyde, product 1) was used directly. After that, product 1 (1.32 g), 2‐chloro‐3‐(hydroxymethylidene) cyclohexene‐1‐carbaldehyde (0.34 g) and sodium acetate (1.65 g, 19.8 mmol) were mixed in 260 mL methanol, and stirred for 1 h at 60°C under N_2_. The solvent was removed by rotary evaporation, and the crude product (product 2) was purified by dissolving in DMF and precipitating in diethyl ether. After filtration, the end‐product, IR‐CF_3_ (0.984 g, 64%), was dried under vacuum. ^1^H NMR (400 MHz, DMSO‐d6) δ 8.43–8.30 (m, 4H), 8.10–8.03 (m, 4H), 7.76 (d, J = 8.4 Hz, 2H), 7.69(t, J = 8.3 Hz, 1H), 7.58‐7.48 (m, 4H), 6.43 (s, 1H), 6.40 (s, 1H), 5.80(s, 4H), 2.63–2.54 (m, 4H), 2.02 (s, 12H), 1.83–1.71 (m, 2H).

### 5.2 Synthesis of Peptides

Peptides were synthesized by Fmoc SPPS strategy. Wang resin was used as the solid phase support. The whole synthesis process was carried out in anhydrous DMF in solid phase peptide synthesis vessels with sieves. In the coupling step, 4 mmol of Fmoc amino acid was used to couple the resin in 5 mL of 0.4 mol L^−1^ NMM in DMF and the coupling time was 40 min. In the deprotection step, 20 v/v % piperidine was used to remove the Fmoc group and the deprotection time was 10 min. Upon completion, a cleavage reagent (95 v/v % TFA, 2.5 v/v % water, and 2.5 v/v % Tips) was added into the vessel for 2 h. The crude peptides were analyzed and purified by high‐performance liquid chromatography system (Waters e2695) with a SunFire C18 column (5 µm, 4.6 mm × 150 mm) at a flow rate of 1.0 mL min^−1^. The column was eluted with a gradient of 5–80% acetonitrile containing 0.1% TFA for 10 min.

### 5.3 Synthesis of PSP@IR‐CF_3_

First, Solution A was prepared by dissolving PSP (2 mg mL^−1^) in PBS (pH 7.4), while Solution B was made by dissolving IR‐CF_3_ in dimethyl sulfoxide (20 mg mL^−1^). Solution A (2 mL) was transferred to a 300 W ultrasonic homogenizer, before 0, 10, 25, 50, 75, and 100 µL of Solution B were subjected to ultrasound treatment. The mixture was sonicated for 2 h using an ultrasonic homogenizer. The mixed solution was then removed and placed in a dialysis bag with a 1000 Da cutoff. After 12 h of dialysis in PBS solution, the liquid was removed from the dialysis membrane and stored in a 4°C refrigerator for incubation. After 24 h, the nanofibers were stably assembled.

### 5.4 Cell Lines

CT26‐Luc and 4T1‐Luc cells (luciferase‐expressing mouse colorectal cancer cells) were sourced from the Key Laboratory of Molecular Imaging, Institute of Automation, Chinese Academy of Sciences (Beijing, China). CT26‐Luc and 4T1‐Luc cells were maintained in a standard medium with 10% fetal bovine serum (FBS; Gibco, Invitrogen) and 100× penicillin–streptomycin solution (Solarbio, Beijing, China). The CT26‐Luc and 4T1‐Luc cells were incubated at 37°C with 21% O_2_ and 5% CO_2_. All cell lines were authenticated by STR profiling and tested negative for mycoplasma contamination.

### 5.5 Animals

BALB/c mice and NTG mice (NOD‐scid IL2Rγ ^−/−^), both 6 weeks old, were obtained from SPF Biotechnology Co. Ltd. (Beijing, China). Animals were kept in a specific pathogen‐free (SPF) environment at 20 ± 3°C with a 12 h light/dark cycle and free access to food and water. Animal procedures followed the guidelines for ethical research use set by Peking Union Medical College Hospital (permit number: XHDW‐2026‐132‐1). Tumor‐bearing mice were monitored daily and were euthanized when tumor volume reached the humane endpoint (≥2000 mm^3^) or when signs of distress, including severe weight loss, ulceration, or impaired mobility, were observed.

### 5.6 Establishment of Subcutaneous Mouse Model

BALB/c mice were injected in their flanks with 1 × 10^6^ CT26‐Luc or 4T1‐Luc cells per mouse to generate the subcutaneous tumor model. In vivo PTIT was performed on CT26‐Luc or 4T1‐Luc tumor‐bearing mice, which were randomly allocated to seven groups: (i) PBS, (ii) IR‐CF_3_ (675 µg mL^−1^, i.v.), (iii) aPD‐L1 (2 mg/mL, i.v.), (iv) PSP@IR‐CF_3_ (675 µg/mL, i.v.), (v) NIR (1 W/cm^2^, 10 min), (vi) IR‐CF_3_ + NIR, (vii) PSP@IR‐CF_3_ + NIR. Bilateral CT26‐Luc tumor‐bearing mice received treatment with PBS or PSP@IR‐CF_3_, with or without NIR, as described previously.

### 5.7 Establishment of PDX Model

PDX tumor specimens were obtained from Sun Yat‐sen Memorial Hospital under the approval of the Institutional Review Board (IRB No. SYSEC‐KY‐KS‐2023‐106). Written informed consent was obtained from all patients prior to sample collection. PDX tumor samples were created by transplanting human breast tumor fragments in situ into the right mammary glands of anesthetized NTG mice. The mice were then observed in SPF conditions. Tumor growth was measured two‐dimensionally using calipers (*n* = 5). The formula for calculating the tumor volume (TV) was TV = (width^2^ x length) × 0.5. The tumors were typically passaged when the TV reached 1 cm^3^.

### 5.8 In Vivo Fluorescence Molecular Imaging (FMI)

CT26‐Luc tumor‐bearing mice were allocated into three groups. In the blocking group, 200 µg of aPD‐L1 was administered intraperitoneally 12 h before imaging, and again 1 h before imaging. Mice anesthetized with isoflurane were subjected to FMI following intravenous administration of IR‐808, with or without PSP@IR‐CF_3_, on the IVIS Spectrum system (Perkin Elmer). Images were acquired before injection and at subsequent time points (0.5, 1, 2, 4, 6, 8, 10, 12, 24, and 48 h), using excitation and emission wavelengths of 745 and 800 nm, respectively. 2 d after injection, the tumor‐bearing mice from each group were euthanized to retrieve the major organs (heart, liver, spleen, lung, kidney, and tumor) for imaging of the excised tissues. In this experiment, aPD‐L1 was employed as a blocking agent to verify the specificity of PSP@IR‐CF_3_ binding and to examine its influence on tumor targeting.

### 5.9 In Vivo Bioluminescence Imaging (BLI)

Bilateral CT26‐Luc tumor‐bearing mice were monitored using BLI. Mice were anesthetized with 1.5% isoflurane in oxygen and intraperitoneally injected with D‐luciferin (150 µg g^−1^ body weight; Perkin Elmer) 8 min before imaging. BLI images were acquired with the IVIS Spectrum system to monitor tumor signals at different time points after treatment in each group (0, 4, 8, 12, and 16 d).

### 5.10 In Vivo Photothermal Conversion

CT26‐Luc or 4T1‐Luc tumor‐bearing mice were allocated to three groups (PBS, IR‐CF_3,_ and PSP@IR‐CF_3_) according to the average tumor volume and were injected intravenously with 100 µL of IR‐CF_3_ or PSP@IR‐CF_3_ at the same concentration of 675 µg mL^−1^. Tumors that were subjected to localized injections of PBS were used as a control group. All tumors were then irradiated with an 808 nm laser (1.0 W/cm^2^, 10 min). During irradiation, the IR images of the mice were obtained with a compact thermal imaging camera (FLIR E60). After treatment, the data including tumor volumes and body weights were collected at predetermined time intervals.

### 5.11 Evaluating Anti‐Tumor Efficacy in the Tumor Model

The anti‐tumor effects of nanofibers were evaluated in CT26‐Luc and 4T1‐Luc tumor‐bearing mice. Once the tumor volumes reached around 80 mm^3^, the mice were randomly allocated into seven groups: PBS (control), IR‐CF_3_, aPD‐L1, PSP@IR‐CF_3_, NIR, IR‐CF_3_ + NIR, and PSP@IR‐CF_3_ + NIR. Mice in the IR‐CF_3_ and IR‐CF_3_ + NIR groups received an intravenous injection of 100 µL IR‐CF_3_ (675 µg mL^−1^). Mice in the PSP@IR‐CF_3_ and PSP@IR‐CF_3_ + NIR groups were injected intravenously with 100 µL PSP@IR‐CF_3_ (IR‐CF_3_: 675 µg mL^−1^) per mouse. NIR irradiation (1.0 W/cm^2^, 10 min) was performed 24 h after injection. Mice in the aPD‐L1 group received an intravenous injection of 100 µL aPD‐L1 (2 mg mL^−1^). Two methods were employed to determine tumor volume. One method used the formula (length × width^2^)/2, whereas the other utilized the bioluminescence intensity determined by the imaging system mentioned above. ICD expression following treatment was assessed by analyzing cleaved caspase‐3, CRT, and HMGB1 levels in tumor regions using immunohistochemistry and immunofluorescence. In this experiment, aPD‐L1 was included as an immunotherapeutic control to compare its antitumor efficacy with that of PSP@IR‐CF_3_ and PSP@IR‐CF_3_ + NIR.

### 5.12 Ex Vivo Analysis of T Cells

To perform immune cell analysis by flow cytometry, tumors or spleens from mice subjected to different treatments were harvested and stained in accordance with the provided protocols. In short, tumor cells were blocked with CD16/32 antibody (101320; BioLegend) before being stained with the following antibody combinations: CTLs and Th cells (anti‐CD45‐BB515, anti‐CD3‐APC‐Fire750, anti‐CD4‐APC, anti‐CD8‐PE‐Cy7, anti‐IFN‐γ‐APC, and anti‐Granzyme B‐PE); DCs (anti‐CD45‐BB515, anti‐CD11c‐APC‐Cy7, anti‐CD80‐PE, anti‐CD86‐BV421, and anti‐MHCII‐APC); Treg (anti‐CD45‐BB515, anti‐CD4‐APC, anti‐CD25‐PE, and anti‐FOXP3‐PerCP‐Cy5.5). Finally, monoclonal antibody‐stained cell suspensions were analyzed using a flow cytometer (NL‐CLC3000; Cytek, Fremont, CA, USA) in accordance with standard protocols. For each test, 1 × 10^4^ cells were analyzed. The gating strategies used for flow cytometric analyses were shown in Figure S44.

### 5.13 Statistical Analysis

All data are presented as mean ± standard error of the mean (SEM), as indicated. Statistical analyses were performed using GraphPad Prism version 10.1.2 (GraphPad Software, Inc., San Diego, CA, USA; RRID: SCR_002798). Statistical significance was assessed using one‐way or two‐way ANOVA, as appropriate. For comparisons of tumor recurrence rates, Fisher's exact test was performed. A value of *p* < 0.05 was considered statistically significant. Statistical significance is indicated as follows: *p* < 0.05 (*), *p* < 0.01 (**), and *p* < 0.001 (***).

## Author Contributions

**Y. D**., **H. X**., **W. H**., and **Z. J**. conceptualized and supervised the entire research project. **X. Z**. authored the manuscript. Both **X. Z**. and **Y. D**. engineered and executed the imaging, therapeutic efficacy, and mechanistic studies. **Z. J**. and **X. T**. were responsible for synthesizing the materials and their characterization. **W. Z**. conducted in vivo imaging experiments on small animals. **J. W**. assisted in the collection of samples from these animals. **M. S**. contributed to establishing and maintaining the animal models. **Y. Z**. performed rat blood collection and in vivo metabolic studies. **H. G**. and **C. L**. participated in data analysis and further characterization of properties.

## Funding

This study was financially supported by Beijing Natural Science Foundation (Grants No. 7254548), the National Natural Science Foundation of China (Grants No. U24A20731), Beijing Natural Science Foundation (Grants No. 7252292, 7244524), Beijing Nova Program (Grants No. 20240484650), the National Natural Science Foundation of China (Grants No. 81227901, 81930053, 62027901, 32101153, 82502418), Shandong Provincial Natural Science Foundation (Grant No. ZR2025LZL007), Young Elite Scientists Sponsorship Program by BATSA (BYESS2023244), Emerging Engineering Interdisciplinary‐Young Scholars Project (PKU2024XGK007), Peking University, and Peking University Medicine Sailing Program for Young Scholars' Scientific & Technological Innovation (BMU2024YFJHPY015), the Fundamental Research Funds for the Central Universities. The study sponsors were not involved in the design of the study, the collection, analysis, or interpretation of data, or the writing of the manuscript.

## Conflicts of Interest

The authors declare no conflicts of interest.

## Supporting information




**Supporting File**: advs78434‐sup‐0001‐SuppMat.docx.

## Data Availability

All data generated or analyzed during this study are included in this published article and the Supplementary information or available from the author upon reasonable request. The RNA‐seq data have been deposited in the BIG Sub database under accession number CRA022620.
