# Supplementary material for: Combined Immune‐Peptide Nanofiber with HSP70/AKT/mTOR Axis Blockade Enhances Photothermal Immunotherapy, Inhibiting Tumor Growth and Recurrence
Source: Adv Sci (Weinh). 2026 Oct 25:e78434. Online ahead of print. doi: 10.1002/advs.78434 (PMC13696528; doi:10.1002/advs.78434)
Supplement: Supplementary file 1 [file ADVS-9999-e78434-s001.docx]

**Supporting information**

**Combined immune-peptide nanofiber with HSP70/AKT/mTOR axis blockade enhances photothermal immunotherapy, inhibiting tumor growth and recurrence**

Xinyu Zhang^a, b, #^, Zhenqi Jiang^c, #,^ *, Wenjia Zhang^d, #^, Juncheng Wu^a, b^, Haozhuo Guo^e, b^, Changlei Li^b^, Mingwei Sun^b^, Xiaoying Tang^c^, Yue Zhang^f^, Wei Huang^g,^ *, Huadan Xue^a,^ *, Yang Du^b, h,^ *

a. Department of Radiology, State Key Laboratory of Complex Severe and Rare Diseases, Peking Union Medical College Hospital, Peking Union Medical College and Chinese Academy of Medical Sciences, No. 1 Shuaifuyuan, Dongcheng District, Beijing, 100730, China
b. CAS Key Laboratory of Molecular Imaging, Beijing Key Laboratory of Molecular Imaging, Institute of Automation, Chinese Academy of Sciences, Beijing, 100190, China
c. School Medical Science and Engineering, Beijing Institute of Technology Zhengzhou Academy of Intelligent Technology, Beijing Institute of Technology, No. 5 South Zhongguancun Street, Haidian District, Beijing, 100081, China
d. Department of Radiology, Peking University People's Hospital, No. 11 Xizhimen Nandajie, Xicheng District, Beijing, 100044, China
e. Department of Interventional Radiology, Shengjing Hospital of China Medical University, Shenyang, Liaoning, 110004, China
f. Laboratory Animal Research Facility, National Infrastructures for Translational Medicine, Institute of Clinical Medicine, Peking Union Medical College Hospital, Chinese Academy of Medical Sciences & Peking Union Medical College, Beijing, 100730, China
g. Department of Radiation Oncology, Shandong Cancer Hospital and Institute, Shandong First Medical University, Shandong Academy of Medical Sciences, No. 440 Jiyan Road, Jinan City, Shandong Province, 250117, China
h. University of Chinese Academy of Sciences, Beijing, 100080, China

^#^ These authors contributed equally: Xinyu Zhang, Zhenqi Jiang, Wenjia Zhang

* Correspondence and reprint request:

Zhenqi Jiang, Ph.D., No. 5 South Zhongguancun Street, Haidian District, Beijing, 100081, China. Email: [jiangzhenqi@bit.edu.cn](mailto:jiangzhenqi@bit.edu.cn)

Wei Huang, M.D., No. 440 Jiyan Road, Jinan City, Shandong Province, 250117, China, Email: alvinbird@163.com

Huadan Xue, M.D., No. 1 Shuaifuyuan, Dong Cheng District, Beijing, 100730, China. Email: bjdanna95@hotmail.com

Yang Du, Ph.D., No.95 Zhongguancun East Road, Haidian District, Beijing, 100190, China. Email: [yang.du@ia.ac.cn](mailto:yang.du@ia.ac.cn)

# Supplementary Methods

# Materials and reagents

Peptide NQ40 (“NTKYFYEDQ”) was synthesized by Nanjing TAIYE Co. Ltd. (Nanjing, China). InVivo MAb anti-mouse PD-L1 (B7-H1) was obtained from BioX (#BE0101; Lebanon, NH, USA; RRID: AB_10949073). The primary antibodies used for immunofluorescence staining included anti-calreticulin rabbit pAb (GB112134; RRID: AB_3751504), anti-HMGB1 rabbit pAb (GB11103; RRID: AB_3675277), and anti-HSP70 rabbit pAb (GB11241; RRID: AB_3714562), all sourced from Servicebio (Wuhan, China). The secondary antibodies, Cy3-conjugated goat anti-rabbit IgG (GB21303; RRID: AB_2861435) and Alexa Fluor® 488-conjugated goat anti-rabbit IgG (GB25303; RRID: AB_2910224), were also obtained from Servicebio. For flow cytometric analysis, Calreticulin Recombinant Rabbit Monoclonal Antibody (SU37-03) (MA5-32131, RRID: AB_2809422), HMGB1 Recombinant Rabbit Monoclonal Antibody (SA39-03) (MA5-31967; RRID: AB_2809261), and HSP70 Recombinant Rabbit Monoclonal Antibody (SA0379) (MA5-31961; RRID: AB_2809255) were purchased from Thermo Fisher Scientific (Waltham, MA, USA). Goat anti-Rabbit IgG (H+L) Cross-Adsorbed Secondary Antibody, Alexa Fluor™ 488 (A-11008; Thermo Fisher Scientific; RRID: AB_143165) was used as the secondary antibody for flow cytometric detection. Fluorochrome-conjugated anti-mouse monoclonal antibodies, including BB515 rat anti-mouse CD45 (564590; BD, Franklin Lakes, NJ, USA; RRID: AB_2738857), APC/Fire™ 750 anti-mouse CD3 (100248; BioLegend, San Diego, CA, USA; RRID: AB_2572118), APC anti-mouse CD4 (100412; BioLegend; RRID: AB_312697), PE/Cyanine7 anti-mouse CD8a (100722; BioLegend; RRID: AB_312761), APC/Cyanine7 anti-mouse CD11c (117324; BioLegend; RRID: AB_830649), PE anti-mouse CD80 (104708; BioLegend; RRID: AB_313129), Brilliant Violet 421™ anti-mouse CD86 (105032; BioLegend; RRID: AB_2650895), APC anti-mouse I-A/I-E (107614; BioLegend; RRID: AB_313329), PE anti-mouse/human CD11b (101208; BioLegend; RRID: AB_312791), PE/Cyanine7 anti-mouse Ly-6G/Ly-6C (Gr-1) (108416; BioLegend; RRID: AB_313381), PE anti-mouse CD25 (102007; BioLegend; RRID: AB_312856), PerCP-Cyanine5.5 anti-mouse FOXP3 Monoclonal Antibody (FJK-16s) (45-5773-82; Thermo Fisher Scientific, Waltham, MA, USA; RRID:AB_914351), PE/Cyanine7 anti-mouse/human CD44 (103030; BioLegend; RRID: AB_830787), APC anti-mouse CD62L (104412; BioLegend; RRID: AB_313099), PE anti-mouse CD4 (116005; BioLegend; RRID: AB_313690), APC-anti-mouse IFN-γ (505809; BioLegend; RRID: AB_315403), PE-anti-mouse Granzyme B (168803; BioLegend; RRID: AB_3741249), and TruStain FcX™ (anti-mouse CD16/32) Antibody (101320; BioLegend; RRID: AB_1574975).

## Synthesis of PSP@IR-CF_3_/IR-808(FITC)

First, PSP@IR-CF_3_ was lyophilized at -80°C to obtain a freeze-dried powder. Then, the powder was re-dissolved in PBS (4 mg/ml). Next, 1 ml of the resulting solution was taken and 10 μl of a DMSO solution (containing IR-808 (FITC) at 20 mg/ml) was added, followed by stirring at room temperature overnight. Afterwards, ultrafiltration (3kDa, 3000g, 30 min) was performed to remove unloaded IR-808 (FITC), resulting in PSP@IR-CF_3_/IR-808(FITC).

## Characterization of PSP@IR-CF_3_ nanofibers

SEM images were taken on Tescan Mira 3 XH (Tescan, Czech). TEM and high-resolution TEM (HR-TEM) images were taken on a FEI Talos F200X G2 (Thermo Fisher, U.S.). Ultraviolet-visible (UV-Vis) absorption spectra were acquired using a UV-vis spectrophotometer (Lambda 950, Perkin Elmer, U.S.). AFM images were taken on a Dimension XR Fast Scan (Bruker, Germany). The DLS and zeta potential were measured at room temperature using a particle size analyzer (Nano-ZS, Malvern, England). Circular dichroism was measured by Chirascan VX (Applied Photophysics, England).

## Molecular dynamics experimental procedure

According to the molar ratios, we used Packmol software to construct the box containing four peptide molecules and two ligand molecules. The system was placed in a cubic box using the gmx editconf command. All MD simulations were executed using GROMACS 2018.4 software, with the Amber99SB force field and SPCE water model.

First, the system was positioned at the center of a cubic box with a side length of 7 nm and randomly filled with water molecules. Appropriate ions replaced some water molecules to ensure the electroneutrality of the system. Energy minimization was then performed using the steepest descent method to optimize the system to its lowest energy state. Subsequently, constrained MD simulations were conducted with ensembles having a constant number of particles, volume, and temperature (NVT) and a constant number of particles, pressure, and temperature (NPT) to equilibrate the system. These steps primarily aim to optimize the interactions between the molecules, solvents, and ions, thereby achieving an optimal simulation system.

The Verlet leapfrog algorithm was used to solve Newton’s equations of motion, with a time step of 2 fs. The Lennard–Jones function was used to calculate the van der Waals forces with a non-bonded cutoff distance of 1.2 nm. The LINCS algorithm constrained all atomic bond lengths, whereas the long-range electrostatic interactions were computed using the particle mesh Ewald method with a grid spacing of 0.16 nm. Periodic boundary conditions were applied in the simulations.

All MD simulations were conducted in an NPT ensemble at 300 K and 1 atm, controlled using the V-rescale and Parrinello–Rahman methods, respectively. The temperature and pressure coupling constants were set to 0.1 and 0.5 ps, respectively. The molecular dynamics simulation lasted for 100 ns.

## Photothermal properties of PSP@IR-CF_3_ nanofibers

To investigate the photothermal properties of IR-CF_3_, PSP@IR-CF_3_ was dissolved in ddH_2_O at 50 μg/mL and irradiated with an 808 nm laser at a power density of 1.0 W/cm^2^ for 10 min. To investigate the relationship between the photothermal effects and the nanofiber concentration, 0, 10, 25, 50, and 100 μg/mL IR-CF_3_ and PSP@IR-CF_3_ were irradiated with 1.0 W/cm^2^ for 10 min. To investigate the relationship between the photothermal effects and laser power, 50 μg/mL IR-CF_3_ and PSP@IR-CF_3_ were irradiated with 0.5, 1.0, 1.5, and 2.0 W/cm^2^ for 10 min. Three individual measurements were performed for each sample, and the mean was calculated.

## Flow cytometry analysis of cellular binding

Flow cytometry was performed to assess the cellular binding of PSP@IR-CF_3_/FITC. Tumor cells were seeded in 6-well plates and treated with PBS, free FITC, non-targeted peptide/FITC, PSP@IR-CF_3_/FITC with aPD-L1 blocking, or PSP@IR-CF_3_/FITC alone. After incubation, cells were washed three times with cold PBS to remove unbound material, detached using EDTA-free trypsin, and resuspended in PBS containing 2% fetal bovine serum (FBS).

Fluorescence signals were measured using a BD FACSCanto II flow cytometer (BD Biosciences), and a minimum of 10,000 cells were recorded per sample. Data were analyzed with FlowJo software v10.8.1 (RRID: SCR_008520), and mean fluorescence intensity (MFI) was used to quantify binding levels.

## Evaluation of *in vitro* targeting specificity

CT26-Luc cells were seeded (approximately 1 × 10^5^ cells/well) in 20 mm glass-bottom dishes for 24 h. Afterwards, aPD-L1 was added to the medium of the blocking groups to a final concentration of 0.3 mg/mL, and then the medium of all groups was replaced with 2 mL of fresh medium containing 100 μg/mL PSP@IR-CF_3_/IR-808 or IR-808. After 4 h, the cells were washed with cold PBS, fixed with 4% paraformaldehyde, and stained with DAPI (Solarbio, Beijing, China). The samples were protected from light throughout this process. Images were obtained using a confocal laser-scanning microscope (LSM780; Carl Zeiss, Jena, Germany).

## Immunogenic cell death (ICD) expression assays

Immunofluorescence staining was used to investigate calreticulin (CRT) overexpression. The CT26-Luc cells were treated with PBS, CF_3_, and PSP@IR-CF_3_ for 12 h, respectively. The cells were exposed to an 808 nm laser (1 W/cm^2^, 10 min) and then incubated for an additional 24 h. Subsequently, the cells were washed with PBS, fixed using 4% formaldehyde, and treated with 3% bovine serum albumin. The rabbit anti-CRT antibody at a 1:100 dilution was applied and left to incubate overnight at 4 °C. After three washes with phosphate buffered saline with Tween-20 (PBST), the cells were stained with a Cy3-conjugated goat anti-rabbit secondary antibody at a 1:300 dilution, and DAPI was used to stain the nuclei. The fluorescence microscope (Nikon, Japan) was used to measure the expression level of CRT.

Overexpression of HMGB1 was investigated with an enzyme-linked immunosorbent assay (ELISA) kit (SEA399Mu 96T, Cloud-clone, Wuhan, China). The CT26-Luc cells were treated with PBS, IR-CF_3,_ and PSP@IR-CF_3_ for 12 h, respectively. The cells were exposed to an 808 nm laser (1 W/cm^2^, 10 min) and then incubated for an additional 24 h. The supernatant was spun at 4 °C for 15 min at 3000 RPM and used immediately for the experiment. HMGB1 release was measured using an HMGB1 ELISA kit following the provided instructions.

## Flow cytometric analysis of ICD-associated markers

CT26-Luc cells were subjected to the indicated treatments and collected for flow cytometric analysis. CRT, HMGB1 and heat shock protein (HSP70) were detected using Alexa Fluor 488-conjugated antibodies according to the manufacturer's instructions. After staining, cells were analyzed by flow cytometry. The resulting data were analyzed using FlowJo software (version 10.8.1).

## Western blotting (WB)

The cells were lysed using lysis buffer containing a protease inhibitor cocktail. The extracts from cells or tissues were denatured and analyzed using sodium dodecyl sulfate polyacrylamide gel electrophoresis (SDS-PAGE). The proteins were then separated by SDS-PAGE and transferred to poly(vinylidene difluoride) (PVDF) membranes. The blots were blocked with 5% nonfat dry milk in tris buffered saline with Tween 20 (TBST). Next, they were exposed to primary antibodies. Signals were detected using horseradish peroxidase (HRP)-conjugated secondary antibodies and the enhanced chemiluminescence (ECL) detection method.

## *In vitro* dendritic cell maturation assay

CT26-Luc cells were subjected to the indicated treatments and subsequently co-cultured with dendritic cells under standard culture conditions. After co-culture, dendritic cells were collected, washed with PBS, and stained with fluorophore-conjugated antibodies against CD80 (CD80-PE) and CD86 (CD86-BV421) according to the manufacturer's instructions. The expression of CD80 and CD86 was analyzed by flow cytometry, and dendritic cell maturation was evaluated based on the percentage of CD80⁺CD86⁺ cells. Flow cytometry data were analyzed using FlowJo software (version 10.8.1).

## RNA sequencing (RNA-seq) analysis

RNA sequencing (RNA-seq) was performed by Novogene (Beijing, China) using an Illumina sequencing platform. Differentially expressed genes (DEGs) among the PSP@IR-CF₃ + NIR, PSP@IR-CF₃ and PBS groups were identified using DESeq2. Kyoto Encyclopedia of Genes and Genomes (KEGG) pathway enrichment analysis was performed to investigate the biological pathways associated with the DEGs. Gene expression patterns were visualized using volcano plots and heatmaps.

## HSP70 knockdown

Short hairpin RNA (shRNA) targeting mouse Hspa1b (HSP70) was designed and constructed by OBiO Technology (Shanghai, China). The shRNA sequence (5′-GCTGACGAAGATGAAGGAGAT-3′) was cloned into the pSLenti-U6-shRNA-CMV-EGFP-F2A-Puro-WPRE vector, with a non-targeting shRNA serving as a negative control. The recombinant plasmids were confirmed by DNA sequencing. Cells were subsequently transfected with the Hspa1b-targeting shRNA plasmid or the corresponding control plasmid. HSP70 knockdown efficiency was evaluated by Western blotting.

## Colony formation assay

CT26 cells were harvested and prepared as a single-cell suspension. Cells were seeded at low density in culture dishes and randomly assigned to five groups: Control, VER-155008, PSP@IR-CF_3_ + NIR, PSP@IR-CF_3_ + NIR + VER-155008, and PSP@IR-CF_3_ + NIR + Rapa. Following the indicated treatments, cells were cultured under standard conditions (37 °C, 5% CO₂) for 14 days until visible colonies had formed.

The culture medium was then removed, and the cells were gently washed with PBS. Colonies were fixed with 4% paraformaldehyde for 10–15 min and stained with crystal violet for 10–20 min. Excess dye was removed by washing with distilled water, and the plates were air-dried. Colonies were quantified using ImageJ software (RRID:SCR_003070).

## Cytokine detection

TNF-α, IL-4, IFN-γ, and IL-6 (all from Multisciences Biotech, Hangzhou, China) in mouse serum were measured with ELISA kits following the standard protocols.

## Safety assessment

Serum and tissue samples were collected after treatment and observation. ALT, AST, and BUN levels were measured with the Automated Chemistry Analyzer (Rayto, Shenzhen, China) following the provided instructions. Histopathological analysis was performed on the heart, liver, spleen, lungs, kidneys, and skin, which were collected and stained with hematoxylin and eosin.

## *In vivo* recurrence study with HSP70 inhibition

CT26-Luc cells were cultured under standard conditions and harvested during the logarithmic growth phase. BALB/c mice were subcutaneously inoculated with CT26-Luc cells to establish tumor-bearing models. When tumors reached the predetermined size, mice were randomly assigned to two groups (n = 10 per group): (1) PSP@IR-CF_3_ + NIR and (2) PSP@IR-CF_3_ + NIR + VER-155008.

For treatment, PSP@IR-CF_3_ was administered to tumor-bearing mice when tumor volumes reached approximately 50 mm^3^. At 24 hours after PSP@IR-CF_3_ administration, tumors were exposed to NIR irradiation to induce PTIT. In the combination group, the HSP70 inhibitor VER-155008 was administered via intraperitoneal injection at a dose of 20 mg kg⁻¹ d⁻¹ for 7 consecutive days beginning immediately after NIR irradiation. Tumor growth was monitored regularly, and mice were followed for 28 days after treatment to evaluate tumor recurrence. Tumor recurrence was defined as the reappearance of measurable tumor growth at the original treatment site following initial tumor regression.

## *In vivo* recurrence study with mTOR inhibition

CT26-Luc cells were cultured under standard conditions and harvested during the logarithmic growth phase. BALB/c mice were subcutaneously inoculated with CT26-Luc cells to establish tumor-bearing models. When tumors reached the predetermined size, mice were randomly assigned to two groups (n = 20 per group): (1) PSP@IR-CF_3_ + NIR and (2) PSP@IR-CF_3_ + NIR + Rapamycin.

For treatment, PSP@IR-CF_3_ was administered to tumor-bearing mice when tumor volumes reached approximately 50 mm^3^. At 24 hours after PSP@IR-CF_3_ administration, tumors were exposed to NIR irradiation to induce PTIT. In the combination group, Rapamycin was administered via intraperitoneal injection at a dose of 2 mg kg⁻¹ d⁻¹ for 7 consecutive days beginning immediately after NIR irradiation. Tumor growth was monitored regularly, and mice were followed for 28 days after treatment to evaluate tumor recurrence. Tumor recurrence was defined as the reappearance of measurable tumor growth at the original treatment site following initial tumor regression.

# Supplementary Figures and Tables

Supplementary Fig. 1 Mass spectrometry (MS) results for PSP.

Supplementary Fig. 2 High performance liquid chromatography (HPLC) results for PSP.

Supplementary Table 1. HPLC analysis results showing retention time, peak height, peak area, and concentration for each chromatographic peak.

───────────────────────────────────────────────────

Peak No	Ret Time	Height	Area	Conc.

───────────────────────────────────────────────────

1	9.243	2391.425	16934.598	0.1252

2	9.395	850918.250	13329848.000	98.5131

3	9.395	56436.730	184262.281	1.3618

───────────────────────────────────────────────────

Total	100.00

Supplementary Fig. 3 ^1^H-NMR for IR-CF_3_.

Supplementary Fig. 4 Liquid chromatography–mass spectrometry (LC-MS) for IR-CF_3_.

Supplementary Fig. 5 SEM images of PSP nanofibers formed through co-assembly with varying amounts of IR-CF_3_.

Supplementary Fig. 6 Atomic Force Microscope (AFM) images of PSP nanofibers formed after co-assembly with different amounts of IR-CF_3_, with the average height of the fibers shown at the bottom of the images.

Supplementary Fig. 7 Transmission electron microscope (TEM) of PSP@IR-CF_3._

Supplementary Fig. 8 Dynamic light scattering (DLS) particle size distribution diagram of PSP@IR-CF_3_ dispersed in DMEM (10% FBS) complete culture medium.

Supplementary Fig. 9 The interaction between the nanofibers and PD-L1 protein.

Supplementary Fig. 10 Construction of PSP@IR-CF_3_ systems.

Supplementary Fig. 11 Energy analysis of dynamic processes.

Supplementary Table 2. Overview of interaction forces, including van der Waals energy, electrostatic energy, polar solvation energy, SASA energy, and binding energy.

| **Forces** | | **Energy (kJ/mol)** |
| --- | --- | --- |
| van der Waal energy | -222.509 ± 73.875 | |
| Electrostattic energy | -277.396 ± 86.734 | |
| Polar solvation energy | 141.342 ±72.455 | |
| SASA energy | -26.088 ± 7.832 | |
| Binding energy | -384.65 ± 92.265 | |

Supplementary Fig. 12 Monitoring temperature changes of PSP@IR-CF_3_ under varying laser power irradiation (n=3).

Supplementary Fig. 13 SEM images of non-targeted peptide nanofibers formed via co-assembly with IR-CF_3_.

Supplementary Fig. 14 Representative flow cytometry analysis of CRT, HMGB1, and HSP70 expression in tumor cells after the indicated treatments (n=3).

Supplementary Fig. 15 Representative flow cytometry plots and quantitative analysis of mature DCs (CD80⁺CD86⁺) in different treatment groups (n=3). One-way ANOVA followed by Tukey's multiple-comparison test was used for statistical analysis. The data are presented as means ± standard error of the mean (SEM). A value of P < 0.05 was considered statistically significant. Statistical significance is indicated as follows: P < 0.05 (*), P < 0.01 (**), and P < 0.001 (***).

Supplementary Fig. 16 Fluorescence imaging and fluorescence intensity of PBS, IR-808, and PSP@IR-CF_3_/IR-808. (a) Fluorescence imaging of PBS, IR-808, and PSP@IR-CF_3_/IR-808. (b) Statistical analysis of fluorescence intensity (n=3). Unpaired two-tailed Student’s t-test was used for comparisons between two groups. The data are presented as means ± SEM. A value of P < 0.05 was considered statistically significant. Statistical significance is indicated as follows: P < 0.05 (*), P < 0.01 (**), and P < 0.001 (***).

Supplementary Fig. 17 Tumor weights of mice in each group after different treatments (n=5). One-way ANOVA followed by Tukey's multiple-comparison test was used for statistical analysis. The data are presented as means ± SEM. A value of P < 0.05 was considered statistically significant. Statistical significance is indicated as follows: P < 0.05 (*), P < 0.01 (**), and P < 0.001 (***).

Supplementary Fig. 18 Body weights of mice in each group after different treatments (n=5).

Supplementary Fig. 19 Assessment of liver (a, b) and renal (c, d) functions in mice treated with PBS, IR-CF_3_, aPD-L1, PSP@IR-CF_3_, PBS + NIR, IR-CF_3_ + NIR, or PSP@IR-CF_3_ + NIR (n=3).

Supplementary Fig. 20 H&E staining of major organs after treatment with PBS, IR-CF_3_, aPD-L1, PSP@IR-CF_3_, PBS + NIR, IR-CF_3_ + NIR, or PSP@IR-CF_3_ + NIR. Scale bars: 50 μm.

Supplementary Fig. 21 In vivo PTIT of PSP@IR-CF_3_ in 4T1-Luc tumor-bearing mice. (a) Representative images of mice at different time points with different treatments. (b-i) Statistical analysis of BLI intensities in different treatment groups (n=5). Two-way ANOVA was used for statistical analysis. The data are presented as means ± SEM. A value of P < 0.05 was considered statistically significant. Statistical significance is indicated as follows: P < 0.05 (*), P < 0.01 (**), and P < 0.001 (***).

Supplementary Fig. 22 H&E, Ki67, and TUNEL staining of tumors in each group. Scale bars: 20 μm.

Supplementary Fig. 23 ICD induced by PTIT of PSP@IR-CF_3_ in vivo in 4T1-Luc tumor-bearing mice. Immunofluorescence staining was used to detect ICD markers CRT and HMGB1. Scale bars: 20 μm.

Supplementary Fig. 24 Flow cytometric analysis of MHC II expression on CD11c⁺ dendritic cells. (a) Representative flow cytometry histograms showing MHC II expression on CD11c⁺ dendritic cells. (b) Quantification of MHC II expression based on geometric mean fluorescence intensity (gMFI) (n=3). One-way ANOVA followed by Tukey's multiple-comparison test was used for statistical analysis. The data are presented as means ± SEM. A value of P < 0.05 was considered statistically significant. Statistical significance is indicated as follows: P < 0.05 (*), P < 0.01 (**), and P < 0.001 (***).

Supplementary Fig. 25 Flow cytometric analysis of CD8⁺ T-cell activation and effector function. (a) Representative flow cytometry dot plots showing the proportion of CD44⁺CD62L⁻ activated/effector cells among CD8⁺ T cells. (b) Representative flow cytometry histograms showing IFN-γ expression among CD8⁺ T cells. (c) Representative flow cytometry histograms showing Granzyme B expression among CD8⁺ T cells. (d–f) Quantitative analysis of the percentages of CD44⁺CD62L⁻ cells among CD8⁺ T cells (d), IFN-γ⁺ cells among CD8⁺ T cells (e), and Granzyme B⁺ cells among CD8⁺ T cells (f). One-way ANOVA followed by Tukey's multiple-comparison test was used for statistical analysis. The data are presented as means ± SEM (n = 3). A value of P < 0.05 was considered statistically significant. Statistical significance is indicated as follows: P < 0.05 (*), P < 0.01 (**), and P < 0.001 (***).

Supplementary Fig. 26 The immunological response of tumors in 4T1-Luc tumor-bearing mice. (a) CD3 expression on the surface of T cells. (b) Populations of CD8^+^ and CD4^+^ T cells. (c) CD80 and CD86 expression on the surface of dendritic cells (DCs). (d) Proportion of mature DCs (n=5). (e) Proportion of CD3^+^ T cells (n=5). (f) Proportion of cytotoxic T lymphocyte (CTL) cells among CD3^+^ T cells (n=5). (g) The proportion of helper T (Th) cells among CD3^+^ T cells (n=5). One-way ANOVA followed by Tukey's multiple-comparison test was used for statistical analysis. The data are presented as means ± SEM. A value of P < 0.05 was considered statistically significant. Statistical significance is indicated as follows: P < 0.05 (*), P < 0.01 (**), and P < 0.001 (***).

Supplementary Fig. 27 Representative tumor recurrence images (a) and tumor volume measurements (b).

Supplementary Fig. 28 Venn diagram of differentially expressed genes in the PBS, PSP@IR-CF_3_ and PSP@IR-CF_3_ + NIR group.

Supplementary Fig. 29 The volcano plot of differentially expressed genes between PSP@IR-CF_3_ + NIR and PSP@IR-CF_3_ group.

Supplementary Fig. 30 Genome-related pathway enrichment of upregulated genes in PSP@IR-CF_3_ + NIR vs. PSP@IR-CF_3_ groups.

Supplementary Fig. 31 Genome-related pathway enrichment of downregulated genes in PSP@IR-CF_3_ + NIR vs. PSP@IR-CF_3_ groups.

Supplementary Fig. 32 Genome-related pathway enrichment of upregulated genes in PSP@IR-CF_3_ vs. PBS groups.

Supplementary Fig. 33 Genome-related pathway enrichment of downregulated genes in PSP@IR-CF_3_ vs. PBS groups.

Supplementary Fig. 34 Original membrane images corresponding to the Western blot (WB) figures. The original images of all membrane sections used for the presented blots are provided in the Supporting Information. Target proteins and loading controls were detected from different molecular weight regions of the same original membrane. The membrane was horizontally sectioned according to molecular weight, while the original lane order and relative positions were preserved. No lanes from different blots or independent experiments were combined.

(a) Original WB data corresponding to Fig. 8f.

(b) Original WB data for proteins other than HSP70 shown in Fig. 8g.

(c) Original WB data for HSP70 protein shown in Fig. 8g.

Supplementary Fig. 35 Quantification of Western blot results shown in Fig. 8f and Fig. 8g. (a) Quantification of protein expression levels in Fig. 8f. (b) Quantification of protein expression levels in Fig. 8g. The band intensities were quantified using ImageJ software and normalized to the corresponding loading controls (n=3). Data are presented as mean ± SEM. Statistical significance was determined using a two-tailed unpaired Student’s t-test. A value of P < 0.05 was considered statistically significant. Statistical significance is indicated as follows: P < 0.05 (*), P < 0.01 (**), and P < 0.001 (***).

Supplementary Fig. 36 Colony formation assay evaluating the clonogenic survival of tumor cells following different treatments. Representative images of colony formation are shown for the Control (upper left), VER-155008 (0.2 μM) (upper middle), PSP@IR-CF_3_ + NIR (upper right), PSP@IR-CF_3_ + NIR + VER-155008 (0.2 μM) (lower left), and PSP@IR-CF_3_ + NIR + Rapa (10 nM) (lower middle) groups. Quantitative analysis of colony numbers is presented in the lower right panel (n=3). One-way ANOVA followed by Tukey's multiple-comparison test was used for statistical analysis. The data are presented as means ± SEM. A value of P < 0.05 was considered statistically significant. Statistical significance is indicated as follows: P < 0.05 (*), P < 0.01 (**), and P < 0.001 (***). Rapamycin: Rapa.

Supplementary Fig. 37 Representative BLI images of the subcutaneous mouse CT26-Luc tumor models at different time points after PSP@IR-CF_3_ + NIR (n=20).

Supplementary Fig. 38 Representative BLI images of the subcutaneous mouse CT26-Luc tumor models at different time points after PSP@IR-CF_3_ + NIR + Rapa (n=20). Rapa, rapamycin.

Supplementary Fig. 39 Therapeutic efficacy of the combination treatment with PSP@IR-CF_3_ + NIR and rapamycin in the subcutaneous mouse CT26-Luc tumor models. (a) Statistical analysis of BLI signal intensities of the subcutaneous mouse tumor models at different time points after different treatments (n=20). (b) Tumor volume measurements in the subcutaneous CT26-Luc mouse models on Day 28 (n=20). Rapa, rapamycin. Unpaired two-tailed Student’s t-test was used for comparisons between two groups. The data are presented as means ± SEM. A value of P < 0.05 was considered statistically significant. Statistical significance is indicated as follows: P < 0.05 (*), P < 0.01 (**), and P < 0.001 (***).

Supplementary Fig. 40 Representative BLI images of the subcutaneous mouse CT26-Luc tumor models at different time points following the indicated treatments (n=10).

Supplementary Fig. 41 Quantification of tumor recurrence rates in CT26-Luc tumor-bearing mice following the indicated treatments.

Supplementary Table 3. Therapeutic efficacy of different treatment groups. Tumor recurrence rates were analyzed using Fisher’s exact test (6/20 vs. 0/20, P = 0.0202).

| Group | Total | Cure | Curative ratio |
| --- | --- | --- | --- |
| PBS | 5 | 0 | 0 |
| PSP@IR-CF_3_ | 5 | 0 | 0 |
| PSP@IR-CF_3_ + NIR | 5 | 3 | 60% |
| PSP@IR-CF_3_ + NIR + Rapa | 5 | 5 | 100% |

Supplementary Fig. 42 H&E staining of major organs after treatment with PBS, PBS + NIR, PSP@IR-CF_3_ + NIR, or PSP@IR-CF_3_ + NIR + Rapa. Scale bars: 50 μm.

Supplementary Fig. 43 Assessment of liver (a, b) and renal (c, d) functions in mice treated with PBS, PBS + NIR, PSP@IR-CF_3_ + NIR, or PSP@IR-CF_3_ + NIR + Rapa (n=3).

Supplementary Fig. 44 Gating strategies used for flow cytometry cell sorting. (a) Gating strategy for the analysis of the indicated single marker. (b) Gating strategy to sort matured DCs (CD11c^+^CD80^+^CD86^+^). (c) Gating strategy to sort CD8^+^ (CD45^+^CD3^+^CD8^+^) T cells and CD4^+^ (CD45^+^CD3^+^CD4^+^) T cells. (d) Gating strategy to sort Tregs (CD4^+^CD25^+^Foxp3^+^).
